# Long-term COVID-19 vaccine- and Omicron infection-induced humoral and cell-mediated immunity

**DOI:** 10.3389/fimmu.2024.1494432

**Published:** 2024-11-21

**Authors:** Milja Belik, Arttu Reinholm, Pekka Kolehmainen, Jemna Heroum, Sari Maljanen, Eda Altan, Pamela Österlund, Larissa Laine, Olli Ritvos, Arja Pasternack, Rauno A. Naves, Alina Iakubovskaia, Alex-Mikael Barkoff, Qiushui He, Johanna Lempainen, Paula A. Tähtinen, Lauri Ivaska, Pinja Jalkanen, Ilkka Julkunen, Laura Kakkola

**Affiliations:** ^1^ Institute of Biomedicine, University of Turku, Turku, Finland; ^2^ Microbiology Unit, Finnish Institute for Health and Welfare, Helsinki, Finland; ^3^ Department of Physiology, Medicum, University of Helsinki, Helsinki, Finland; ^4^ InFlames Research Flagship Center, University of Turku, Turku, Finland; ^5^ Department of Paediatrics and Adolescent Medicine, Turku University Hospital and University of Turku, Turku, Finland; ^6^ Clinical Microbiology, Turku University Hospital, Turku, Finland

**Keywords:** COVID-19, mRNA vaccination, T cell responses, B cell responses, booster vaccination, cell-mediated immunity, long term immunity, hybrid immunity

## Abstract

**Introduction:**

Mutations occurring in the spike (S) protein of SARS-CoV-2 enables the virus to evade COVID-19 vaccine- and infection-induced immunity.

**Methods:**

Here we provide a comprehensive analysis of humoral and cell-mediated immunity in 111 healthcare workers who received three or four vaccine doses and were followed up to 12 and 6 months, respectively, after the last vaccine dose. Omicron breakthrough infection occurred in 71% of the vaccinees, enabling evaluation of vaccine- and vaccine/infection-induced hybrid immunity.

**Results:**

Neutralizing antibodies were the highest against the ancestral D614G and were sequentially reduced against the Omicron variants BA.2, BA.5 and XBB.1.5. S1-specific IgG and neutralizing antibody levels were significantly higher in infected than in uninfected vaccinees, and the fourth vaccine dose in combination with a breakthrough infection resulted in high neutralizing antibody levels against all variants. T cell-mediated immunity, instead, was well retained already after two vaccine doses, and was not significantly strengthened by additional booster vaccine doses or Omicron breakthrough infections.

**Discussion:**

While humoral immunity is sensitive to mutations in the S protein and thus declined rapidly, the cell-mediated immunity is durable to antigenic variation, which may explain the good efficacy of COVID-19 vaccines against a severe disease.

## 1 Introduction

The ongoing evolution of the Severe Acute Respiratory Syndrome Coronavirus 2 (SARS-CoV-2) leads to the emergence of novel virus variants that can evade vaccine and infection-induced immunity. Since November 2021 the Omicron variants (1) have been circulating globally causing infections, reinfections, as well as breakthrough infections among the vaccinees despite the introduction of the Omicron variant-specific vaccines (2, 3). Regardless of the progress made in vaccine development, questions remain on the effectiveness of current vaccines against emerging variants, on the need for booster doses to enhance vaccine-induced responses and to override immune imprinting, and on the effect of infection-induced hybrid immunity.

Humoral immunity has received significant attention, and it has been shown that vaccines and infections induce circulating antibodies which, however, wane with time (4, 5). Even though breakthrough infections induce broadly neutralizing antibodies, the neutralization capacity against emerging variants wanes (6, 7). In addition to humoral immunity, cell-mediated immunity is strongly induced by the SARS-CoV-2 vaccines and infections. It has been shown that T-cell responses elicited by both infections and vaccinations are long-lasting and cross-reactive between virus variants (8–11), emphasizing the importance of cell-mediated immunity in the protection against a severe disease. With the growing number of vaccinees encountering breakthrough infections (12), it is necessary to fully understand the role of long-term cell-mediated immunity in vaccine- and infection-induced immune responses.

In this study, we investigated the humoral and T- and B-cell-mediated immune responses to the ancestral SARS-CoV-2 and Omicron variants BA.2, BA.5, and XBB.1.5, among Finnish healthcare workers. In Finland, the administration of the third vaccine dose began in September 2021 and the fourth dose in April 2022. We followed the immune responses in the vaccinees up to 12 months after the third dose and six months after the fourth dose. We show that during the Omicron era, breakthrough infections were very common resulting in higher IgG antibody levels in the infected vaccinees. Both SARS-CoV-2 infection and the fourth vaccine dose elicited neutralizing antibodies, however, the capacity to neutralize emerging variants was lower compared to the original variant. In contrast, spike-specific CD4+ T-cell responses were similar in vaccinees with or without hybrid immunity. Our study provides a detailed analysis of the long-term immune responses post the third and the fourth vaccine doses, and on vaccine- and infection-induced hybrid immunity. This information is crucial for the decision makers in deciding the recommendations on COVID-19 vaccine compositions and booster dose administrations.

## 2 Materials and methods

### 2.1 Ethical statement

The study was approved by Southwest Finland health district Ethical Review Board (ETMK 19/1801/2020, EudraCT 2021-004419-14). Study participants signed a written informed consent before the first sampling.

### 2.2 Serum samples and peripheral blood mononuclear cells

In this study, altogether 111 participants (**Table 1**) were included from a cohort of health care workers (HCWs) of the Turku University Hospital who were recruited before the start of COVID-19 vaccination program (13). The vaccinees received two sequential doses of COVID-19 vaccine with a 3-week or a 12-week interval, followed by a third dose 3–9 months later. HCWs belonging to risk groups for severe COVID-19 (except the participants with severe and moderate disorders of the immune system) received the fourth vaccine dose 4-23 months after the third dose. ChAdOx1, BNT162b2, or mRNA-1273 were given as the first dose, and BNT162b2 or mRNA-1273 as the second and third doses. The fourth vaccine dose was either the original mRNA vaccine or a bivalent BA.1 or BA.4/5 vaccine. Breakthrough infections were identified based on self-reporting by the study participants (positive SARS-CoV-2 antigen or RT-qPCR test result), and/or based on an increase greater than the cut-off value in anti-S1 (4.8 EIA units) or anti-N (8.8 EIA units) IgG antibody levels.

**Table 1:** Demographics of the study participants.

	All	3D	4D
N	111	74	37
Female (%)	106 (95.5%)	70 (95.9%)	36 (94.7%)
Male (%)	5 (4.5%)	4 (5.4%)	1 (4.5%)
Infected (%)	79 (71.2%)	64 (86.5%)	15 (40.5%)
Double infected (%)	10 (9.0%)	8 (10.8%)	2 (5.4%)
Age in years			
Mean	47.2	43.2	55.3
Median	49	43	60
Range	23–66	23–64	28–66
SARS-CoV-2 infections* by time point			
Pre–2D5mo	2	2	0
2D5mo–3D3wk	3	3	0
3D3wk–3D3mo	30	28	2
3D3mo–3D6mo	18	12	6
3D6mo–3D8mo	2	2	0
3D8mo–3D12mo	28	25	3
3D12mo–4D3wk	2	0	2
4D3wk–4D3mo	2	0	2
4D3mo–4D6mo	2	0	2
All time points	89	72	17
Average number of months between vaccine doses and infections* (range)			
Between the 2nd and 3rd doses	5.8 (2.7–9.4)	5.7 (4.4–6.8)	6.1 (2.7–9.4)
Between the 3rd and 4th doses	9.7 (4.3–14.1)	–	9.7 (4.3–14.1)
Between infection and previous vaccine dose or infection	6.1 (0.2–12.8)	6.2 (0.2–12.8)	5.8 (0.4–12.4)
Average number of months between infection* and subsequent sampling (range)			
Pre–2D5mo	0.4 (0.4–0.4)	0.4 (0.4–0.4)	–
2D5mo–3D3wk	0.6 (0.5–0.7)	0.6 (0.5–0.7)	–
3D3wk–3D3mo	1.4 (0.3–3.1)	1.4 (0.3–3.1)	2.1 (1.5–2.8)
3D3mo–3D6mo	1.8 (0.4–4.3)	1.8 (0.4–4.3)	1.7 (1.3–2.3)
3D6mo–3D8mo	1.2 (0.8–1.6)	1.2 (0.8–1.6)	–
3D8mo–3D312mo	2.3 (0.3–5.5)	2.1 (0.3–5.5)	3.9 (3.4–4.7)
3D12mo–4D3wk	0.5 (0.5–0.5)	–	0.5 (0.5–0.5)
4D3wk–4D3mo	0.6 (0.6–0.6)	–	0.6 (0.6–0.6)
4D3mo–4D6mo	0.9 (0.5–1.3)	–	0.9 (0.5–1.3)
All time points	1.7 (0.3–5.5)	1.7 (0.3–5.5)	1.8 (0.5–4.7)

*SARS-CoV-2 infections at each time point were confirmed using PCR, antigen testing, or S1- and N-specific enzyme immunoassays. Infections confirmed by PCR or antigen testing were included in the calculations for the intervals between infections, vaccinations, and sample collections.

### 2.3 SARS-CoV-2 variants

The SARS-CoV-2 isolates used in this study were FIN25-20 (Pango lineage B.1, D614G strain, GenBank ID MW717675.1 and GISAID ID EPI_ISL_412971), FIN58-22 (lineage B.1.1.529.2, Omicron BA.2 variant, OP199045 and EPI_ISL_9695067), FIN61-22 (lineage B.1.1.529.5, Omicron BA.5 variant, OP199047 and EPI_ISL_13118918) and FIN69-22 (Omicron XBB.1.5 variant, OQ509907 and EPI_ISL_16526646). To isolate the SARS-CoV-2 variants, SARS-CoV-2 PCR-positive nasopharyngeal samples were inoculated to VeroE6 (for D614G) or VeroE6-TMPRSS2-H10 cells (14) (for Omicron BA.2, BA.5, and XBB.1.5) and passaged in VeroE6-TMPRSS2-H10 cell in DMEM (EuroClone) supplemented with 2% fetal calf serum (FCS; Gibco), 2mM L-glutamine (Gibco), and penicillin-streptomycin. Tissue Culture Infectious Dose (TCID50) assay was used to determine virus stock titers as described previously (15, 16).

### 2.4 SARS-CoV-2 S1- and N-specific enzyme immunoassay

SARS-CoV-2 S1- and N-specific IgG antibody levels in sera were measured with an in-house immunoassay (EIA), as described previously (15, 16). Briefly, purified recombinant SARS-CoV-2 S1 (3.5 μg/ml) and N (2.0 μg/ml) proteins were coated on 96-well immunoplates. Sera were diluted (1:1,000 for S1-specific EIA, 1:300 for N-specific EIA), and antigen-specific IgG levels were determined by measuring the absorbance at 450 nm. The optical density (OD) values obtained were converted to EIA units using a linear interpolation between OD-values of known positive (100 EIA units) and negative (0 EIA units) serum specimens. The thresholds to determine seropositivity were established as described previously (15, 16).

### 2.5 Microneutralization tests

The titers of neutralizing antibodies in the sera were determined with a microneutralization test (MNT) as described previously (15, 16). Briefly, starting from a 1:5 dilution, a two-fold dilution series was prepared for each serum sample into DMEM supplemented with 2% FCS, penicillin-streptomycin, 2 mM L-glutamine. Subsequently, 50 TCID_50_ of virus was added, resulting in serum dilutions from 1:10 up to 1:40,960 (D614G) or 1:2,560 (Omicron variants). Virus-serum dilution mixtures were incubated at +37 °C, 5% CO_2_ for 1 h before adding VeroE6-TMPRSS2-H10 cells (45) (50,000 cells per well in 96-well plate). After incubation for 4 days at +37°C, 5% CO_2_, the cells were fixed with 4% formaldehyde, stained with crystal violet, and visualized for cell death. Reciprocal of the serum dilution able to inhibit 50% of cell death was considered as the neutralization titer (half-maximal inhibitory dose, ID50). ID50>10 was considered positive for neutralizing antibodies. Controls (positive control serum with neutralizing antibodies, cells alone, and virus without serum) were included in every MNT plate.

### 2.6 ELISpot for SARS-CoV-2 specific memory B cells

To detect the circulating memory B cells capable of maturing into antibody-secreting cells, PBMCs were stimulated for five days with 1 μg/ml R848 (TLR7/8 agonist; InvivoGen), 0.01 μg/ml recombinant IL-2 (R&D systems), and 10% FCS in RPMI-1640 medium. ELISpot plates (multiscreen filtration plate, Millipore) were coated overnight at +4°C with his-tagged SARS-CoV-2 N (8 μg/ml), S1 (3.5 μg/ml), and RBD (4 μg/ml) proteins, and with mouse myostatin (8 μg/ml; a control for nonspecific binding) (16), tetanus toxoid, Td (10 μg/ml; GlaxoSmithKline), and unlabeled anti-human IgG (10 μg/ml; MP Biomedicals). The plates were blocked for 1h with AIM-V medium (Thermo scientific) supplemented with 10% FCS and 50mM of 2-mercaptoethanol (AIM-V+ medium) and washed. Subsequently, antigen-specific amount of stimulated PBMCs in AIM-V+ medium was added to the plates: 500,000 cells to SARS-CoV-N and mouse myostatin, 100,000 cells to tetanus toxoid and RBD, 50,000 cells to S1, and 10,000 cells to anti-IgG coated wells, all in duplicates. After 24 h incubation, the wells were washed with PBS/0.5% Tween-20, and the cells were lysed with distilled water. The cell debris was washed off with PBS/0.5% Tween-20, and alkaline phosphatase-conjugated goat anti-human IgG (Sigma-Aldrich) was added for 1h, +37°C. The plates were washed with PBS/0.5% Tween-20, allowed to dry, and washed thrice with PBS before adding 1-Step NBT/BCIP-substrate (Pierce) for 5 min. The plates were washed and allowed to dry before imaging and analysis with ImmunoScan C.T.L ELISpot reader. Nonspecific spots (mouse myostatin spots) were subtracted from the antigen-stimulated spots for each sample. Samples without specific spots for both anti-IgG and tetanus toxoid (11/105) were excluded from the analysis.

### 2.7 Activation induced marker assay and flow cytometry

SARS-CoV-2 S- and N-specific T cells were detected with activation induced marker (AIM) assay as a frequency of CD134+CD69+CD4+ or CD137+CD69+CD8+ T cells among stimulated PBMCs as previously described (17). Briefly, cryopreserved PBMC were thawed and resuspended in culture media (RPMI-1640, Lonza) supplemented with 10% heat-inactivated human AB serum (Sigma), 2mM L-glutamine, and penicillin-streptomycin. After washing, cell viability was assessed with TC20 cell counter (Biorad), and 1×10^6^ cells/well were plated in 96-well round-bottom plates (Thermo). Cells were stimulated with overlapping S peptide pools (Pepmix, JPT peptides) covering the entire S protein of the ancestral Wuhan-Hu-1 (here referred as wild type, wt) or Omicron variants BA.5 and XBB.1.5 at 0.5 µg/ml peptide per stimulation, as well as with a wt N peptide pool covering the entire N protein at 1 µg/ml peptide per stimulation (PM-WCPV-S-1, PM-SARS2-SMUT10-1, PM-SARS2-SMUT15-1, and PM-WCPV-NCAP-1, respectively). Incubation with tetanus toxoid (10µg/ml, AJ vaccines) was used as a positive control, and incubation with an equimolar amount of DMSO was used as a negative control. Cells were cultured for 48h at +37°C, 5% CO_2_. After stimulation, cells were washed with staining buffer (PBS supplemented with 2% FCS and 0.01% NaN3), and stained with Zombie Green viability dye (BioLegend, 1:1000) for 15min. Cells were washed and stained with fluorochrome-conjugated anti-CD3 (Invitrogen), anti-CD45 (Invitrogen), anti-CD4 (Invitrogen), anti-CD8 (Invitrogen), anti-CD69 (BD Biosciences), anti-CD134 (Biolegend), anti-CD137 (Biolegend), anti-CD45RA (Biolegend), anti-CCR7 (Biolegend), and anti-CXCR5 (Biolegend) antibodies (**Supplementary Table 2**). After surface staining, cells were washed and resuspended in a staining buffer for acquisition with NovoCyte Quanteon Flow Cytometer (Agilent Technologies Inc) and analyzed with NovoExpress v1.5.0 (Agilent Technologies Inc). A stimulation index (SI) was calculated by dividing the frequency of CD134+CD69+CD4+ or CD137+CD69+CD8+ T cells after SARS-CoV-2 peptide pool stimulation by the frequency of CD134+CD69+CD4+ or CD137+CD69+CD8+ T cells after DMSO stimulation. Samples with <10,000 CD3+ T cells were excluded from all analyses and samples with <500 cTfh CD4+ T cells were excluded from the analysis of activated cTfh cells.

### 2.8 Cytokines

Supernatants from the stimulated PBMCs were analyzed for IFN-γ cytokine levels using the MILLIPLEX Kit HCD8MAG-15K (Millipore) in Turku Center for Disease Modeling. Fluorescence was measured with the Luminex MAGPIX magnetic bead analyzer (Luminex Corporation), and median fluorescent intensity values were calculated for seven diluted standards (included in the MILLIPLEX assay kit) to determine cytokine concentrations via a five-parameter logistic regression. Quantification was performed only for samples within the linear range. For statistical purposes, samples below the lowest standard in the linear range were assigned a value of half of the lowest standard value (0.5 pg/ml), while those exceeding the highest standard in the linear range were assigned the highest standard value (5000 pg/ml). Standard samples were measured in duplicates. According to the manufacturer, samples with fewer than 35 beads per well were considered unreliable and were excluded from the analysis.

### 2.9 Statistics and reproducibility

Raw data from all assays was arranged in Excel 2016 (Microsoft 365), and analyzed and processed into graphs with GraphPad Prism (version 10.1.2). Shapiro-Wilk and Kolmogorov-Smirnov tests were used to test for the normality of the data. For paired samples Wilcoxon signed-rank test or Friedman test followed by Dunn’s multiple comparisons was used, and for unpaired samples or time points where multiple participants lacked samples, Mann Whitney U-test or two-sided Kruskal-Wallis test followed by Dunn’s multiple comparisons was used to test statistical significance. All tests were two-sided and statistically significant (p-value <0.05) differences are presented in figure legends. Correlation between the values was tested using Spearman’s correlation test.

## 3 Results

### 3.1 Demographics of the study participants

**Figure 1:**
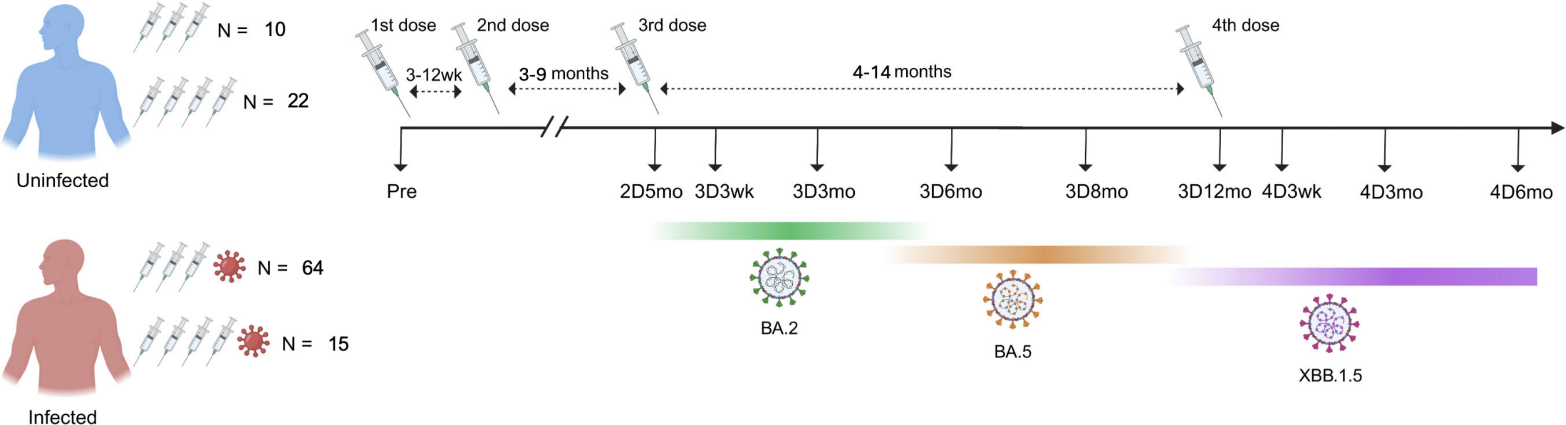
Timeline of blood sample collection from COVID-19 vaccinated participants with and without Omicron infection. Altogether 111 HCWs were included: 32 uninfected (N=10 with three vaccine doses, N=22 with four vaccine doses) and 79 infected (N=64 with three vaccine doses, N=15 with four vaccine doses). The intervals between the second and third doses, as well as between the third and fourth doses, are calculated as averages. Dominant variants circulating during the follow-up period are marked below the timeline. Information about the circulating variant is based on genomic surveillance of SARS-CoV-2 by the Finnish Institute for Health and Welfare.

In our previous studies, we have analyzed serum samples collected from healthcare workers (HCWs) up to nine months after the third COVID-19 vaccine dose (18). To expand the knowledge on long-term vaccine-induced immunity, in this study we included sequential serum samples from 111 HCWs collected from pre-vaccination up to 12 months after the third vaccine dose (N=74) and up to six months after the fourth vaccine dose (N=37; the fourth vaccine dose is recommended only for risk groups) (**Figure 1**). The age of study participants ranged from 23 to 66 years (median 49 years) and the majority were females (96%) (**Table 1**). Those who received four vaccine doses were somewhat older than those who received three vaccine doses (median 60 years versus 43 years, respectively). The fourth vaccine dose was given 4–14 months (median 9.7 months) after the third vaccine dose, and 17 participants received the original monovalent mRNA vaccine and 14 received a bivalent BA.1 or BA.4/5 mRNA vaccine as the fourth vaccine dose. Six participants had no information available on the type of the fourth vaccine dose. The serum samples were collected before any COVID-19 vaccinations (Pre), before the third dose (five months after the second vaccine dose, 2D5mo) and three weeks, and three, six, eight, and twelve months after the third dose (3D3wk, 3D3mo, 3D6mo, 3D8mo, 3D12mo, respectively), as well as three weeks, and three, and six months after the fourth dose (4D3wk, 4D3mo, 4D6mo, respectively) (**Figure 1**).

Of the 111 HCWs, 71% (79/111) had a PCR- or antigen-test confirmed SARS-CoV-2 infection and 9% (10/111) had two consecutive infections (a total of 89 infection events) by the end of the follow-up period (**Table 1**). Two participants had an infection before the first serum sample collection, 78 had an infection after the third dose (0.3–5.5 months before subsequent sampling), and six had an infection after the fourth dose (0.5–1.3 months before subsequent sampling). Most of the breakthrough infections (54%; 48/89) were observed when Omicron BA.2 was spreading in Finland (early January to middle of June 2022, becoming the most prevalent variant in March 2022), and 46% (41/89) of the infections were observed between late June and late December 2022 when Omicron BA.5 was the most prevalent variant (19). Uninfected (no confirmed SARS-CoV-2 infection) vaccinees had similar age distribution compared with the infected vaccinees.

### 3.2 SARS-CoV-2 S1- and N-specific IgG antibody responses in vaccinees with or without a breakthrough infection

**Figure 2:**
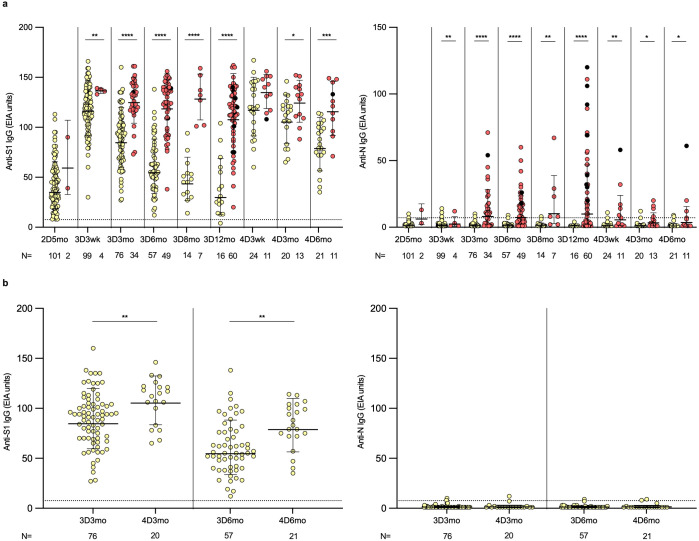
SARS-CoV-2 S1- and N-specific IgG antibody responses in HCWs after two, three and four vaccine doses. **(A)** SARS-CoV-2 S1- and N-specific IgG antibody levels were measured with EIA from the serum samples collected from three and four times vaccinated healthcare workers (HCWs) five months after the second vaccine dose (2D5mo; N=103), three weeks (3D3wk; N=103) and three (3D3mo; N=110), six (3D6mo; N=106), eight (3D8mo; N=21) and twelve months (3D12mo; N=76) after the third dose, and three weeks (4D3wk; N=35), and three (4D3mo; N=33) and six months (4D6mo; N=32) after the fourth dose. Yellow dots refer to uninfected vaccinees, red dots represent samples collected from vaccinees with a SARS-CoV-2 breakthrough infection, and black dots represent samples collected from vaccinees with two (or more) breakthrough infections. **(B)** S1- and N-specific IgG antibody levels compared between three and six months after the third and fourth vaccine doses in uninfected vaccinees. Dashed lines indicate the cut-off values. Geometric means and geometric standard deviations of the IgG antibody levels are shown. Mann-Whitney U-test was used to analyze the statistical significance between the samples collected from uninfected and infected vaccinees within time points, and between the samples collected from uninfected vaccinees three and six months after the third and fourth vaccine dose. Two-tailed p-values <0.05 were considered statistically significant. *p<0.05; **p<0.01; ***p<0.001; ****p<0.0001.

To analyze the humoral immunity against SARS-CoV-2, IgG antibodies were measured in the sera of 111 vaccinees that remained uninfected or experienced breakthrough infections during the follow-up. Five months after the second vaccine dose, the geometric mean (GM) of S1-specific antibody levels was 35 EIA units among vaccinees who had not experienced a breakthrough infection (**Figure 2A**). Following the third vaccine dose, the IgG levels rose to 115 EIA units. Subsequently, over the next twelve months, S1-specific IgG antibody levels declined, returning to levels close to those observed before the third vaccine dose (GM of 30 EIA units at 3D12mo). The decay was faster at three weeks to six months post vaccination (2.1-fold from 3D3wk to 3D6mo) and slowed at six to twelve months post vaccination (1.8-fold from 3D6mo to 3D12mo). Administration of the fourth vaccine dose restored S1-specific IgG antibody levels with levels reaching 117 EIA units at three weeks post the fourth vaccine dose (4D3wk). After the fourth dose, the antibody levels decayed slower compared to that observed after the third dose (1.5-fold from 4D3wk to 4D6mo). Effectively, slower decay rate resulted in S1-specific IgG antibody levels being at higher levels after the fourth dose compared to after the third dose (84 EIA units at 3D3mo and 105 EIA units at 4D3mo, p<0.003; 55 EIA units at 3D6mo and 79 EIA units at 4D6mo, p<0.001) (**Figure 2B**). At the end of the follow-up period (3D12mo N=16 and 4D6mo N=21), all except one uninfected participant had detectable S1-specific IgG antibodies (**Figure 2A**). Of note, the increase in S1-specific IgG antibodies was equally high between the participants vaccinated with monovalent and the bivalent BA.1 or BA.4/5 booster vaccine doses (**Supplementary Figure 1**). N protein-specific IgG antibodies remained under the cut-off value in 98% (99/101) of the uninfected vaccinees throughout the follow-up period (**Figure 2A**).

The results of the uninfected HCWs in combination with our previous results (19, 26) show a trend in the antibody kinetics: the first booster dose markedly elevated the antibody levels (5.2 fold increase three weeks and 7.0 fold increase three months after the second dose compared to the corresponding time points after the first dose), followed by a decline to only 1.0–1.3 fold increase three weeks and three months after the third and fourth doses (**Supplementary Table 1**). However, the third and fourth doses restored the antibody levels at three weeks post vaccination close to the levels observed three weeks after the previous vaccine dose. Interestingly, when comparing the levels at three months post vaccination to the levels at three weeks post vaccination the decay rate of the antibody levels diminished after subsequent vaccine doses (0.4, 0.6, 0.7 and 0.9 fold increase after the first, second, third and fourth vaccine dose, respectively).

Among vaccinees who contracted one or more breakthrough infections, the N-specific IgG antibody levels were relatively low after the first breakthrough infection, and 19/79 (24%) failed to produce detectable levels of anti-N antibodies (**Figure 2A**). Similar to the uninfected vaccinees, in infected vaccinees after the fourth vaccine dose the S1-specific IgG antibody levels increased to comparable levels as was seen after the third vaccine dose (GM 137 EIA units at 3D3wk versus 136 EIA units at 4D3wk). Throughout the follow-up after the third and fourth vaccine doses, S1-specific IgG antibody levels were significantly higher in the infected vaccinees compared to the uninfected vaccinees (except at 4D3wk). These results indicate that constant breakthrough infections boost the antibody production to a high level. This booster effect of infection is evident when infected vaccinees were followed in more detail (**Supplementary Figure 2**). With a few individual exceptions, a consistent trend was observed: S1-specific IgG antibodies were initially induced through vaccination or infection, followed by a relatively similar kinetics of decline.

### 3.3 Neutralizing antibodies against four SARS-CoV-2 variants in vaccinees up to six months after the fourth dose

**Figure 3:**
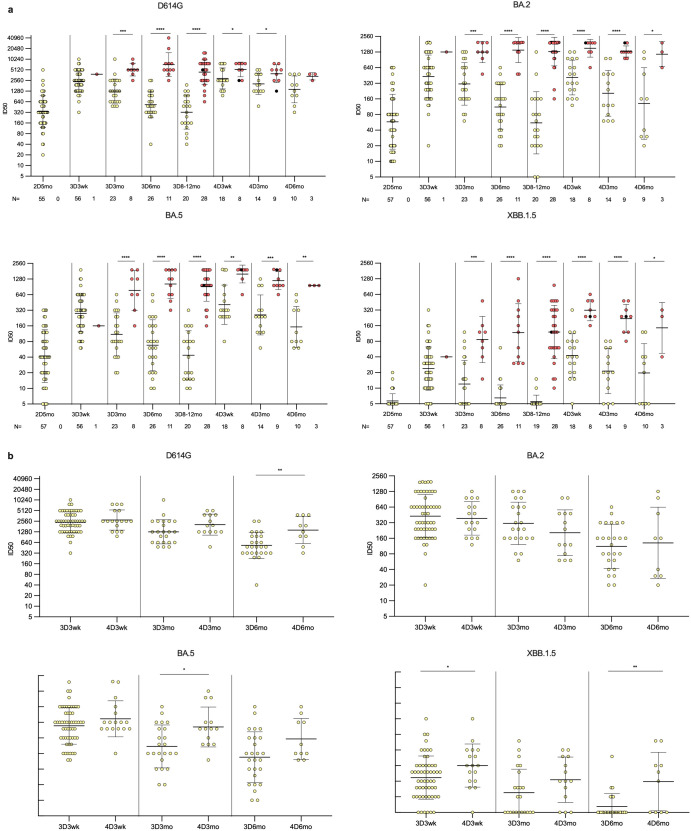
Neutralizing antibody responses against four variants in HCWs up to six months post fourth vaccine dose. **(A)** Neutralizing antibody titers of uninfected (yellow dots) and infected (red dots) HCWs post two, three, and four vaccine doses. Samples were collected from three and four times vaccinated HCWs five months after the second vaccine dose (2D5mo; N=57), three weeks (3D3wk; N=57), and three (3D3mo; N=31), six (3D6mo; N=37), and eight to twelve months (3D8-12mo; N=48) after the third vaccine dose, three weeks (4D3wk; N=26), and three (4D3mo; N=23) and six (4D6mo; N=13) months after the fourth vaccine dose. Neutralizing antibody titers were measured against four SARS-CoV-2 variants: D614G, Omicron BA.2, Omicron BA.5, and Omicron XBB.1.5. **(B)** Comparison of third dose timepoints to the equivalent time points after fourth dose in uninfected vaccinees. Geometric means and geometric standard deviations of the antibody levels are shown. Mann-Whitney U-test was used to analyze the statistical significance between the samples collected from uninfected and infected participants within time points, and between the samples collected from participants three weeks, and three and six months after the third and fourth vaccine dose. Two-tailed p-values <0.05 were considered statistically significant. *p<0.05; **p<0.01; ***p<0.001; ****p<0.0001.

To assess the neutralization capability of antibodies induced by vaccination and Omicron breakthrough infections, neutralizing antibody titers were analyzed using a microneutralization test (MNT) with serum samples collected from 57 participants (**Figure 3**), who were selected in comparable numbers from the groups of infected and uninfected vaccinees and of those who received three (uninfected N=10, infected N=20) or four vaccine doses (uninfected N=15, infected N=11). The infected vaccinees were further selected to equally represent probable infections with the BA.2 (N=12) and BA.5 (N=19) Omicron strains, based on the time of infection confirmed by PCR or antigen test. Neutralization efficiency was tested against four SARS-CoV-2 variants: the ancestral strain D614G and Omicron variants BA.2, BA.5, and XBB.1.5.

Neutralizing antibodies were detected against D614G and Omicron BA.5 in all vaccinees at all time points post the third and fourth vaccine doses (**Figure 3A**). Neutralizing antibodies against Omicron BA.2 were also detected in almost all vaccinees; only two uninfected vaccinees (2/48, 4.2%) had titers below the detection limit at 3D8–12mo time point. However, the levels of neutralizing antibodies against a later Omicron variant, XBB.1.5, were lower, and were not detected in 6/57 vaccinees (11%) at 3D3wk, 11/31 vaccinees (35%) at 3D3mo, 20/37 vaccinees (54%) at 3D6mo, 16/47 vaccinees (34%) at 3D8–12mo, 1/26 vaccinees (4%) at 4D3wk, 3/23 vaccinees (13%) at 4D3mo, and 4/13 vaccinees (31%) at 4D6mo. The infected vaccinees had neutralizing antibodies against all variants at all time points, except for one individual whose titers against XBB.1.5 were below the detection limit at the 3D8–12mo time point. Post the third vaccine dose, the neutralizing antibody titers against all variants were significantly higher in vaccinees with a breakthrough infection compared to uninfected vaccinees.

**Figure 4:**
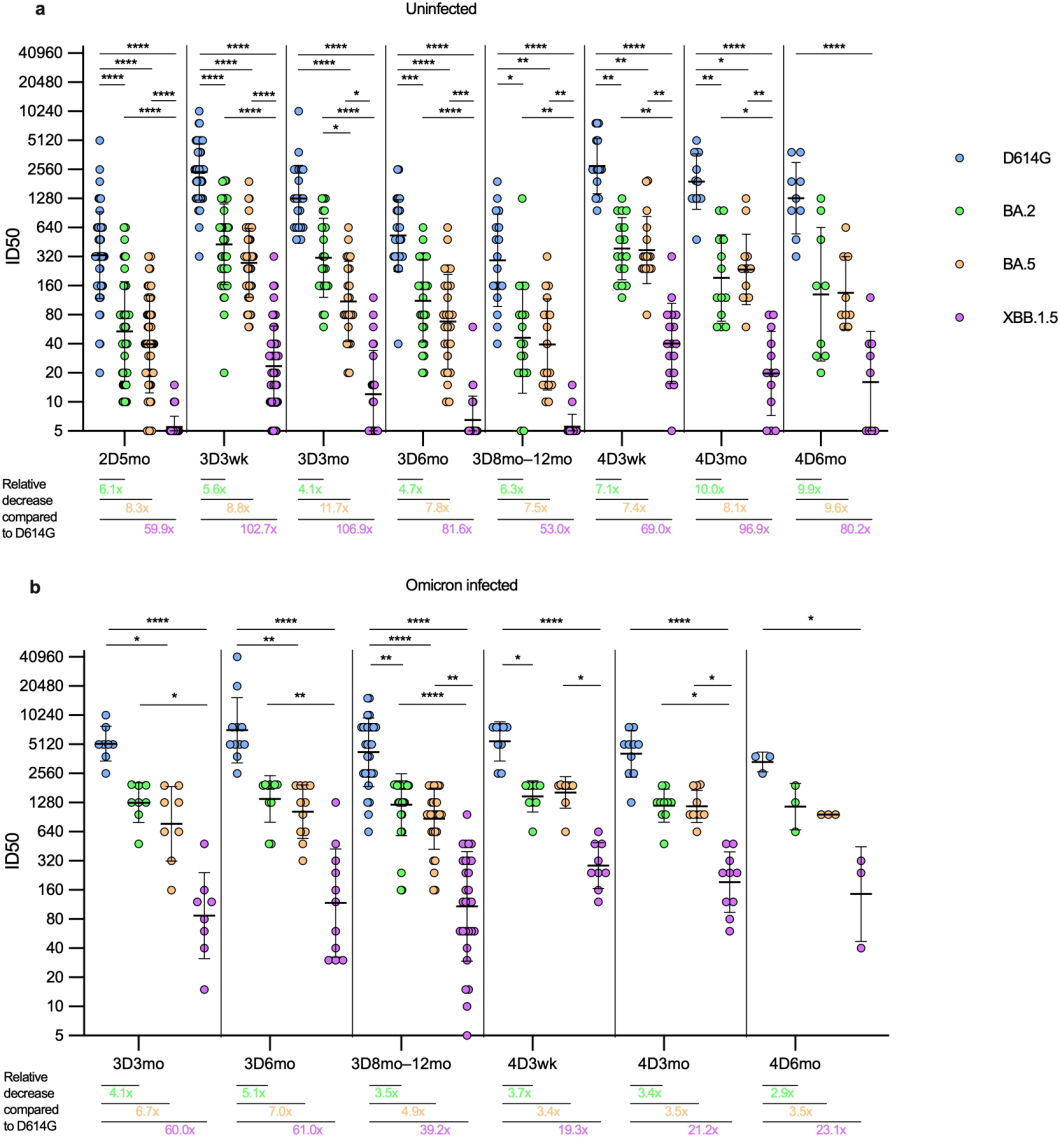
Comparison of neutralizing antibody titers against four SARS-CoV-2 variants in HCWs with and without SARS-CoV-2 infection. Neutralizing antibody titers after two, three, and four vaccine doses against four SARS-CoV-2 variants were compared in uninfected and infected HCWs. **(A)** Samples were collected from three and four times vaccinated and uninfected HCWs five months after the second vaccine dose (2D5mo; N=54), three weeks (3D3wk; N=55) and three (3D3mo; N=23), six (3D6mo; N=26), and eight to twelve months (3D8-12mo; N=18) after the third vaccine dose, three weeks (4D3wk; N=17), and three (4D3mo; N=13) and six (4D6mo; N=9) months after the fourth vaccine dose. **(B)** Samples were collected from three and four times vaccinated HCWs with a breakthrough infection three (3D3mo; N=8), six (3D6mo; N=11), and eight to twelve months (3D8-12mo; N=29) after the third vaccine dose, three weeks (4D3wk; N=9), and three (4D3mo; N=10) and six (4D6mo; N=3) months after the fourth vaccine dose. Neutralizing antibody titers were measured against SARS-CoV-2 variants D614G (yellow dots), BA.2 (green dots), BA.5 (orange dots), and XBB.1.5 (purple dots). Geometric means and geometric standard deviations of the antibody levels are shown. Friedmann test with Dunns’ multiple comparison was used to analyze the statistical significance between the samples collected from uninfected and infected vaccinees within time points, and between the samples collected from vaccinees three and six months after the third and fourth vaccine dose. Two-tailed p-values <0.05 were considered statistically significant. *p<0.05; **p<0.01; ***p<0.001; ****p<0.0001. Fold changes were calculated by comparing the geometric means of the Omicron variants to the D614G at each timepoint.

Comparison of the neutralizing antibody titers in uninfected vaccinees in corresponding time points post the third and fourth vaccine doses revealed relatively similar levels of neutralization titers at three weeks and at three months post vaccinations against D614G and BA.2, whereas for XBB.1.5 and BA.5 there was a significant difference at three weeks and three months post vaccine doses, respectively (**Figure 3B**). Neutralizing antibody titers at six months post the fourth dose were significantly higher than six months post the third dose against D614G and XBB.1.5. Both in uninfected and Omicron infected vaccinees, the neutralizing antibody titers were highest against the ancestral D614G at all time points (**Figures 4A, B**). The neutralization capacity of antibodies decreased with a similar trend for all four variants, with the lowest neutralizing capacity against Omicron XBB.1.5 at all-time points.

In uninfected vaccinees (N=56, **Figure 4A**), there were significant differences in neutralizing antibody titers against the variants at all time points, except between BA.2 and BA.5 the difference was significant only at the 3D3mo time point (p=0.037). In uninfected vaccinees, the fold decrease (compared to D614G) of neutralization titers against Omicron variants ranged between 4.0 to 102.9; the highest fold decreases were observed in the neutralizing titers against XBB.1.5 (fold decrease ranging between 53.0–106.9). In general, in all time points, the fold decrease was higher with evolving Omicron variants, except at three and six months post the fourth dose where the fold decrease was slightly higher for BA.2 than for BA.5 (10.0 versus 8.1 and 9.9 versus 9.6, respectively).

In vaccinees with one or more breakthrough infections (N=27, **Figure 4B**), the difference in neutralizing antibody titers against D614G versus XBB.1.5 was statistically significantly higher in all time points, whereas the difference in neutralizing antibody titers between BA.2 and BA.5 was not significant at any time point. Interestingly, the difference in the geometric mean (GM) of the neutralizing antibody levels against D614G, BA.2, and BA.5 stayed relatively stable regardless of the time point. The neutralization capacity against XBB.1.5 was 5–10 fold lower than against BA.2 and BA.5, i.e. the fold decrease (compared to D614G) of neutralization titers against BA.2 and BA.5 variants ranged between 3.1 to 6.6, whereas against XBB.1.5 the fold decrease of neutralization titers ranged between 19.3 to 62.9. As with the uninfected vaccinees, in all time points the fold decrease was higher with evolving Omicron variants, except three weeks post the fourth dose where the fold decrease was slightly higher for BA.2 than for BA.5 (3.7 vs. 3.4, respectively).

The fourth vaccine dose or an Omicron breakthrough infection resulted in a similar increase in the neutralizing antibody titers against D614G, BA.2 and BA.5 (1.2–1.8 fold increase, **Supplementary Figure 3**) between samples taken pre and post infection and/or vaccination. The fold increase was higher in the neutralizing antibodies against XBB.1.5 after the fourth vaccine dose or a breakthrough infection (2.1 and 3.1, respectively). The fourth vaccine dose in combination with an Omicron breakthrough infection resulted in a higher fold increase in neutralizing antibodies (3.6-8.9) compared to that induced by the fourth vaccine alone against all variants, with the highest fold change in neutralizing antibodies against XBB.1.5. Regardless of the vaccine type, the monovalent and bivalent vaccines as the fourth dose induced similar neutralization titers against all four SARS-CoV-2 variants. There was no significant difference in neutralizing antibody titers against any variant in uninfected vaccinees post monovalent versus bivalent vaccine (**Supplementary Figure 4**).

These results indicate that the antibodies elicited by mRNA vaccines based on ancestral SARS-CoV-2 or bivalent BA.1 or BA.4/5 vaccine neutralize Omicron variants, and the neutralization titers are rescued after each vaccination or a breakthrough infection. However, the neutralization titers, and thus the duration of the neutralization capability, decreases with each evolving Omicron variant.

### 3.4 The functional memory B cell response to vaccinations and SARS-CoV-2 infections

**Figure 5:**
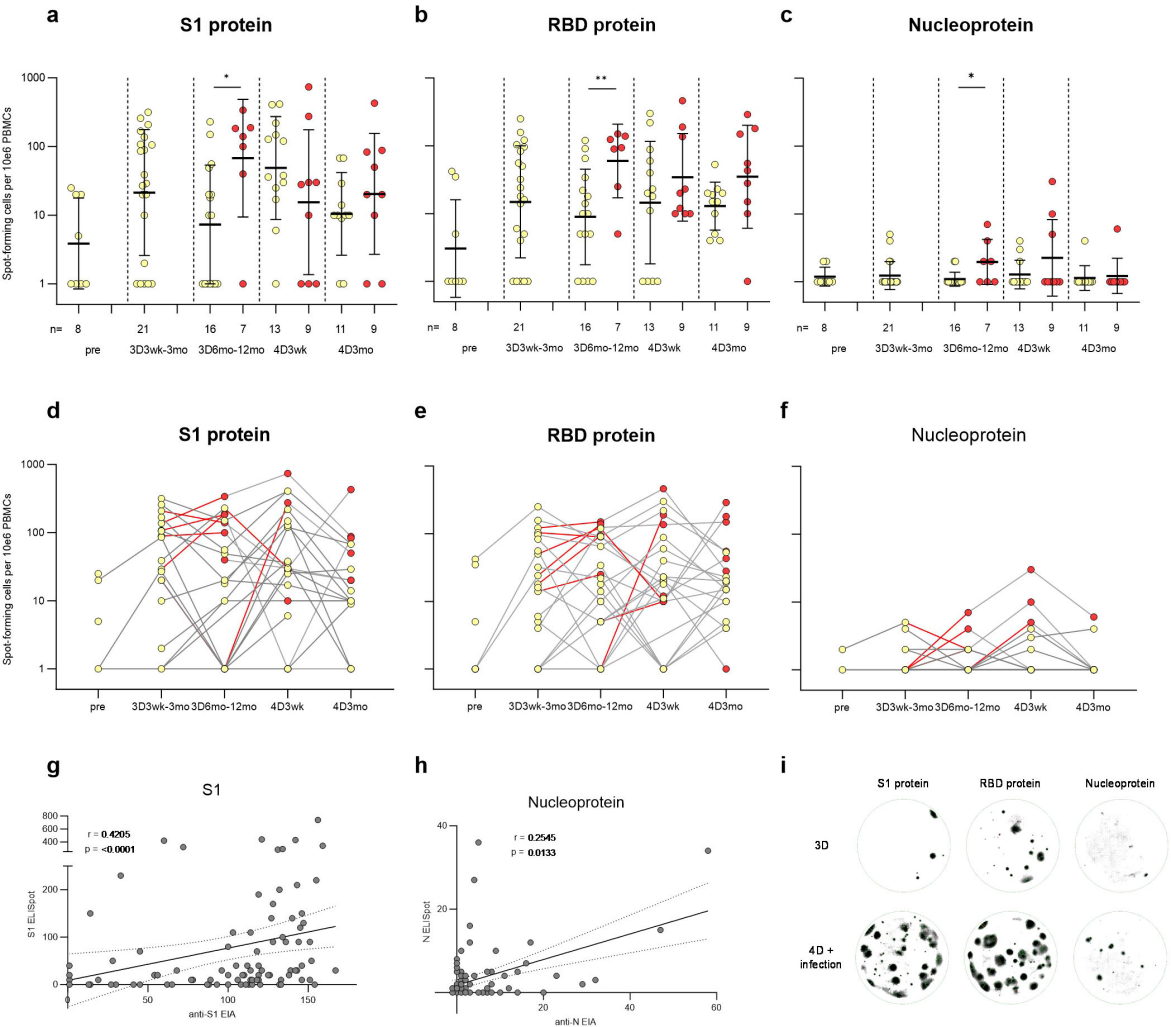
SARS-CoV-2 S1, RBD, and nucleoprotein-specific memory B cell response in vaccinees. PBMCs collected before COVID-19 vaccination (pre, N=8), three weeks or twelve months after the third vaccine dose (3D3wk, N=21; 3D12mo, N=23), and three weeks or three months after the fourth vaccine dose (4D3wk, N=22; 4D3mo, N=20) were stimulated with IL-2 and R848. ELISpot was used to detect the SARS-CoV-2 **(A, D)** S1, **(B, E)** RBD, and **(C, F)** N-specific memory B cells capable of turning into antibody-secreting cells. **(A–F)** Yellow dots indicate spot-forming cells collected from uninfected participants, red dots indicate the spot-forming cells after SARS-CoV-2 infection, and red lines the interval during which a breakthrough infection occurred. Statistical significance was determined using Mann-Whitney U test. **(G–H)** Nonparametric Spearman correlation analysis of the SARS-CoV-2 S1- and N-specific memory B cell responses (S1 and N ELISpot) to S1- and N-specific IgG antibody responses (anti-S1 and anti-N EIA). **(I)** Visual representation of the spots formed by memory B cells specific to S1, RBD, and N antigens in individuals who received three vaccine doses, or four vaccine doses and experienced a breakthrough infection. Two-tailed p-values <0.05 were considered statistically significant. *p<0.05; **p<0.01.

To further characterize humoral immune responses post-vaccination and following a breakthrough infection, the capacity of circulating memory B cells to differentiate into functional antibody-secreting cells (ASCs) was determined with an enzyme-linked immune absorbent spot assay (ELISpot). Antibodies secreted by ASC were assessed against S1, RBD, and N proteins from cultured peripheral blood mononuclear cells (PBMCs) collected from four times vaccinated participants (N=29) (**Figure 5**). Altogether 105 samples from five time points were assayed, and the 94 samples (89.5%) with detectable spots for positive controls (tetanus toxoid and/or IgG) were included in the ELISpot analysis (**Supplementary Figure 5**). Ten (10/29) vaccinees contracted a SARS-CoV-2 breakthrough infection during the follow-up.

Fifty percent of the samples before any COVID-19 vaccinations (pre; 4/8) displayed detectable levels of S1-specific ASC responses (**Figures 5A, D**). Three vaccine doses increased the average number of S1-specific ASCs and 76% (16/21) of the vaccinees had S1-specific ASCs at three weeks to three months after the third dose. The number of S1-specific ASCs declined post vaccination, however, SARS-CoV-2 infection or an additional vaccine dose again increased the number of S1-specific ASCs. RBD-specific ASCs showed a similar trend to that of S1-specific ASCs (**Figures 5B, E**). Vaccinees with a breakthrough infection 6–12 months post the third vaccine dose showed a significantly higher number of S1, RBD, and N-specific ASCs in comparison to uninfected vaccinees (**Figures 5A–C**). The N-specific ACSs were not detected in all SARS-CoV-2 infected vaccinees (**Figures 5C, F**), akin to the levels of infection-induced anti-N IgG antibodies (**Figure 2A**). Nevertheless, the levels of anti-S1 and anti-N IgG antibodies correlated with the corresponding memory B cell responses (r=0.4205, p < 0.0001 for S1; r=0.2545, p = 0.0133 for N; **Figures 5G, H**). In addition, the levels of anti-S1 IgG antibodies correlated with RBD-specific memory B cell responses (r=0.3287, p = 0.0012) (**Supplementary Figures 5F–H**). Overall, the SARS-CoV-2-specific blood-derived functional memory B cell responses were relatively modest. Only some participants with elevated levels of anti-S1 or anti-N antibodies had detectable specific ASCs.

### 3.5 CD4^+^ T cell and circulating T follicular helper cell responses to ancestral and Omicron BA.5 and XBB.1.5 variants

**Figure 6:**
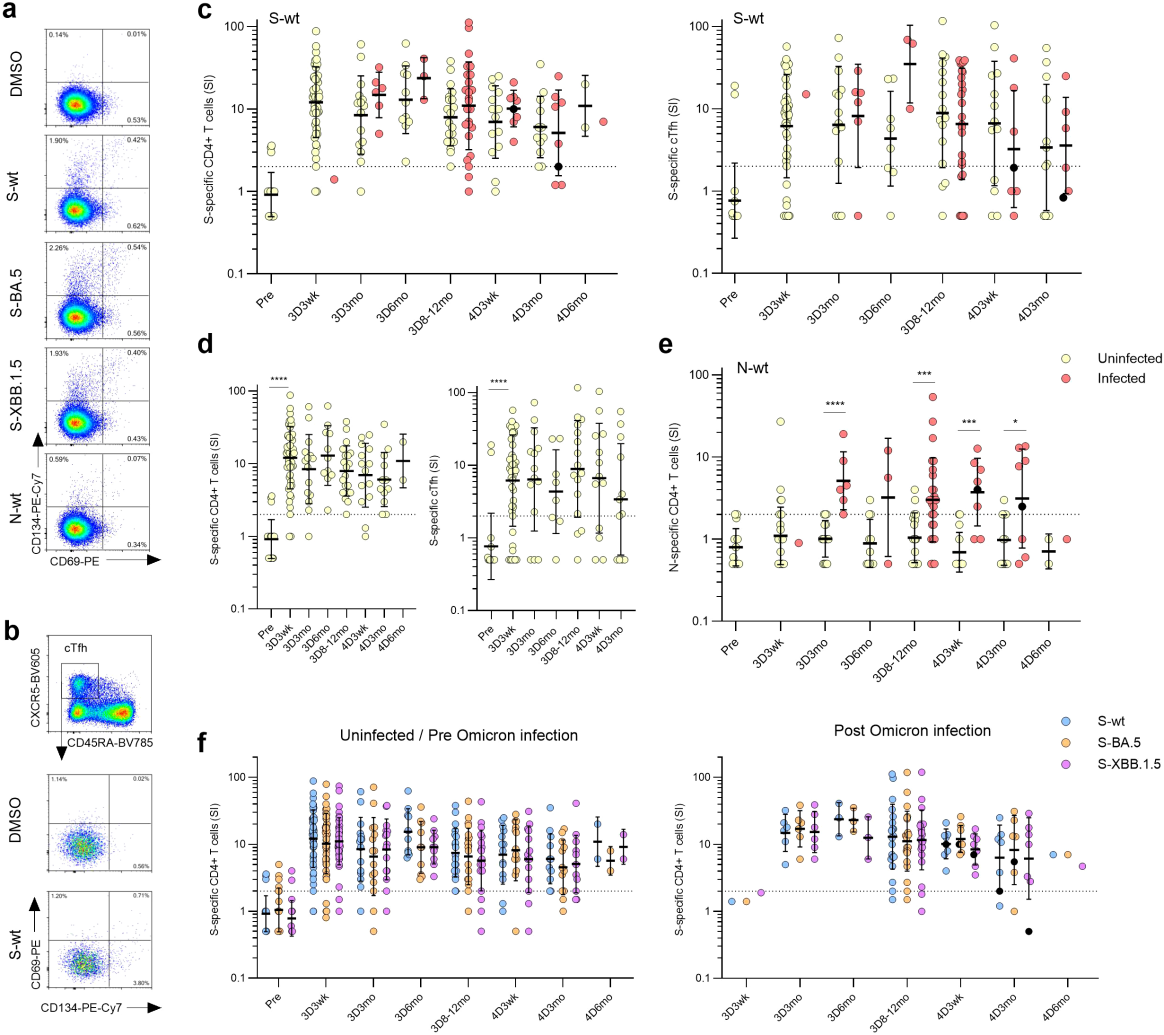
SARS-CoV-2 S-specific CD4+ and circulating follicular T-helper cells (cTfh) in COVID-19 vaccinees. **(A)** Representative flow cytometry plots and gating of CD4+ T cells expressing CD69+CD134+ upon stimulation with DMSO, S or N peptide pools. **(B)** Representative flow cytometry plots of antigen-specific (CD69+CD134+) cTfhs (CD4+CXCR5+CD45RA-) upon stimulation with DMSO or S peptide pool from wild type strain (S-wt). **(C)** S-wt-specific CD4+ and cTfh responses before COVID-19 vaccination (Pre) and after three and four vaccine doses (3D and 4D, respectively). Samples collected after one (red dots) or two breakthrough infections (black dots) were separated at each time point. **(D)** Longitudinal analysis of S-wt-specific CD4+ and cTfh cells in the uninfected vaccinees. **(E)** N-wt-specific CD4+ responses before COVID-19 vaccination and after three and four vaccine doses. **(F)** Comparison of the S-specific CD4+ and cTfh responses after stimulation with S-wt and S peptide pools from Omicron variants BA.5 and XBB.1.5 (S-BA.5 and S-XBB.1.5). Only vaccinees with results from all stimulants were included in the analysis. **(C–F)** Data is shown as geometric means and geometric standard deviations. Mann-Whitney U test was used to analyze differences between uninfected and infected participants and sequential time points. Differences between variant-specific responses were analyzed with a Friedman test followed with Dunn’s multiple comparison test. A two-tailed p<0.05 is considered a significant difference. *p<0.05; ***p<0.001; ****p<0.0001.

To determine the T-cell-mediated immune responses contributing to B cell activation, peripheral blood mononuclear cells (PBMCs) from 55 vaccinees with three (N=29) or four (N=26) vaccine doses were analyzed for CD4+ and T follicular helper cell responses (**Figure 6**). PBMCs were collected before the COVID-19 vaccinations (Pre) and at the same time points as serum samples post the third and fourth vaccine doses. Due to a low number of samples, 3D8mo and 3D12mo time points were grouped into 3D8-12mo time point. PBMCs were stimulated with pooled peptides covering the entire SARS-CoV-2 S protein of original Wuhan Hu-1 (S-wt), Omicron BA.5 or XBB.1.5. During the follow-up period, 55% (30/55) of the vaccinees contracted an Omicron breakthrough infection and one had two consecutive Omicron infections and thus the PBMCs were also stimulated with wt N protein peptides (N-wt). The stimulated PBMCs were analyzed with a flow cytometry for the expression of the activation induced markers (AIM; CD69^+^ and CD134^+^). The gating strategy is described in **Figures 6A, B**, and in **Supplementary Figure 6**.

To quantify the antigen-specific CD4^+^ T cell responses, the frequencies of the CD69^+^CD134^+^ cells stimulated with peptide pools in relation to the cells stimulated with a negative control stimulus (DMSO) were determined. The activation levels of S-wt-specific CD4^+^ T cells remained stable throughout the follow-up (**Figure 6C**, left panel), and 95% of uninfected participants (19/20) showed a positive response still eight months to a year after the last vaccination. In addition, in analysis of samples with sufficient amount of follicular T helper cells (cTfh, CXCR5+CD45RA-, samples with >500 cTfh CD4+ T cells), stable levels of S-wt-specific circulating cTfh cells were detected in most of the participants (34/44, 77% at 3D3wk and 3/16, 81% at 3D8-12mo) (**Figure 6C**, right panel). Notably, the fourth vaccine dose or a breakthrough infection did not significantly increase the frequency of S-wt-specific CD4+ or cTfh cells (**Figure 6C**; **Supplementary Figure 7A**). Among the uninfected vaccinees, the GM of the S-wt-specific CD4+ stimulation indices (SI) remained relatively stable (**Figure 6D**; GM SIs post the third vaccine dose were 12.1, 8.4, 13.0 and 8.0 at 3D3wk, 3D3mo, 3D6mo, and 3D8-12mo, respectively, and post the fourth vaccine dose 7.0, 6.1, and 11.0 at 4D3wk, 4D3mo, and 4D6mo, respectively). Among the infected vaccinees, the GM SIs were 14.8, 23.7 and 11.0 at 3D3mo, 3D6mo, and 3D8-12mo, and 10.2, and 5.1 at 4D3wk and 4D3mo, respectively (**Figure 6C**). There were no significant differences in the GM of the S-wt-specific CD4+ SIs between the uninfected and infected groups at any time point. Breakthrough infections elicited the formation of N-specific CD4+ T cells in 76% (22/29) of participants, and the levels maintained consistent throughout the observation period (**Figure 6E**). CD4+ T cell responses to N peptides were detected also in some participants before the infection (N=11), but the activation levels were significantly lower compared to the levels seen after a breakthrough infection (p<0.0001 at 3D3mo, p=0.0005 at 3D8-12mo, p=0.0001 at 4D3wk, and p=0.0498 at 4D3mo). The S-specific CD4+ and cTfh cell responses in uninfected and Omicron-infected vaccinees were similar upon stimulations with the wt or the Omicron BA.5 or XBB.1.5 peptide pools (**Figure 6F**; **Supplementary Figure 7B**), indicating that the T-cells are cross-reactive for the SARS-CoV-2 variants. Of note, the levels of S-specific cTfh cells did not correlate with S1-specific IgG antibodies (**Supplementary Figure 7C**). The results demonstrate that Omicron-reactive CD4+ T cells are stimulated with both monovalent and bivalent booster vaccines as well as with an Omicron infection.

**Figure 7:**
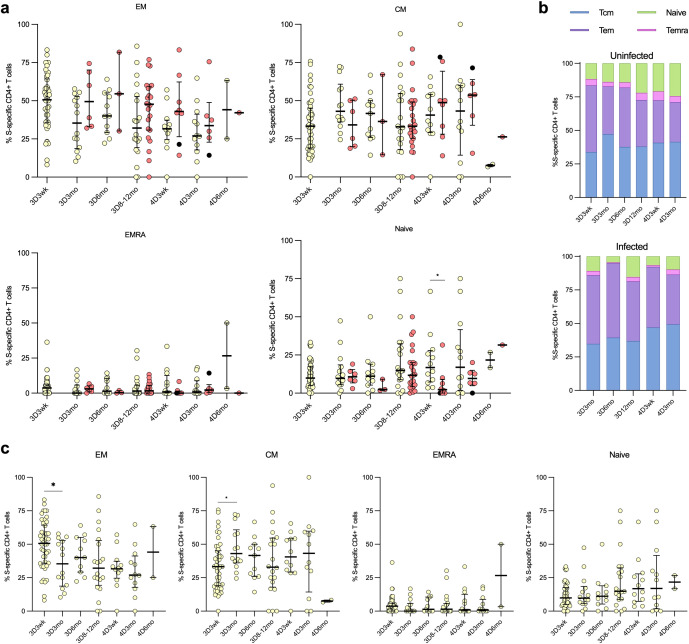
Distribution of the CD4+ cells into memory cell subsets. The distribution of SARS-CoV-2 S-wt-specific CD4+ cells into memory T cell subsets: effector memory (TEM), central memory (TCM), effector memory CD45RA+ (TEMRA), and naïve cells among uninfected and breakthrough-infected vaccinees after three (3D) and four vaccinations (4D). **(A)** Comparison of the memory cell subsets between uninfected (yellow dots) and breakthrough-infected (red dots) vaccinees. **(B)** The memory cell subsets illustrated in stacked bar charts, demonstrating the respective proportions in percentages. **(C)** The memory cell subsets of the uninfected vaccinees are displayed separately. Data is shown as median and interquartile range. Mann-Whitney U test was used to analyze differences between uninfected and infected participants and sequential time points. A two-tailed p<0.05 is considered a significant difference. *p<0.05.

To define the CD4+ T cells contributing to the cell-mediated immune memory, SARS-CoV-2 wt-S-specific CD4^+^ T cells were further analyzed for the distribution into central (Tcm; CD45RA-CCR7-), effector (Tem; CD45RA-CCR7+), terminally differentiated effector (Temra; CD45RA+CCR7-) and naïve-like (CD45RA+CCR7+) memory cell subsets (**Figure 7**). Post the third vaccine dose, the predominant S-specific CD4^+^ memory cell subsets in both uninfected and infected vaccinees were Tcm and Tem cells, with no notable alteration following the administration of the fourth vaccine dose (**Figures 7A, B**). The levels remained constant throughout the follow-up period, with no significant differences between uninfected and infected vaccinees, except for the 4D3wk time point, where uninfected vaccinees showed a higher proportion of naïve cells than infected vaccinees. A marginal, albeit nonsignificant, rise in the proportions of S-specific naïve memory cells and a corresponding decline in Tem cells among uninfected vaccinees was observed across the follow-up period (**Figures 7B, C**).

### 3.6 CD8+ T cell responses to ancestral and Omicron BA.5 and XBB.1.5 variants

**Figure 8:**
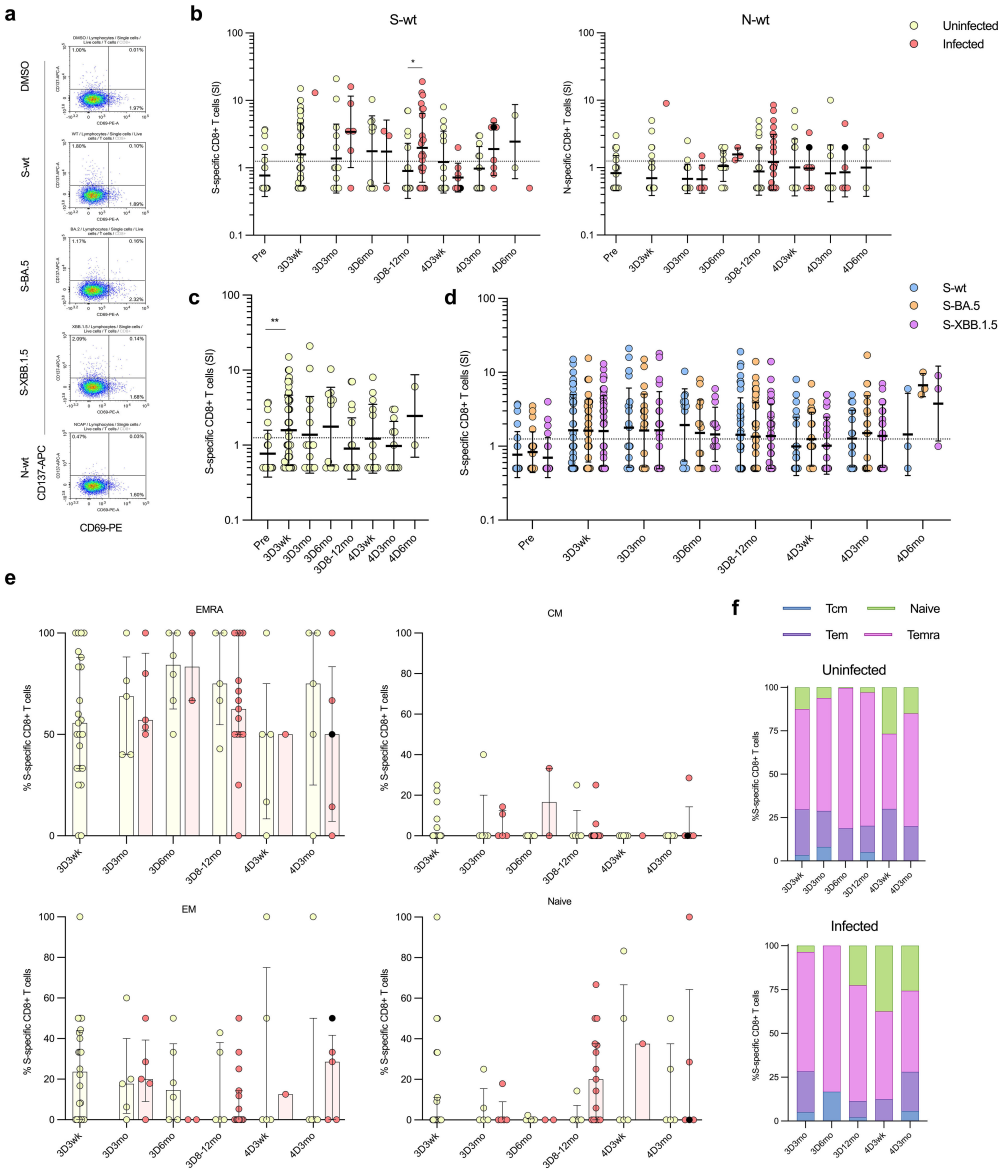
SARS-CoV-2 S-specific CD8+ T cells in COVID-19 vaccinees. **(A)** Representative flow cytometry plots and gating of CD8+ T cells expressing CD69+CD137+ upon stimulation with DMSO or S or N peptide pool (S-wt or N-wt, respectively). **(B)** S-wt and N-wt-specific CD8+ responses before COVID-19 vaccination (Pre) and after three and four vaccine doses (3D and 4D, respectively). Samples collected after one (red dots) or two breakthrough infections (black dots) are separated at each time point. **(C)** Longitudinal analysis of S-wt-specific CD8+ cells in uninfected vaccinees. **(D)** Comparison of the S-specific CD8+ cell responses at different time points after stimulation with S-wt and S peptide pool of Omicron variants BA.5 and XBB.1.5 (S-BA.5 and S-XBB.1.5). Results from uninfected and infected vaccinees are shown together, and only vaccinees with results from all stimulants are included in the analysis. **(E)** The distribution of the S-wt-specific CD8+ cells into memory subsets effector memory (EM), central memory (CM), terminally differentiated effector memory (TEMRA), and naïve cells among both uninfected and breakthrough-infected vaccinees. **(F)** The memory cell subsets are illustrated in stacked bar charts, demonstrating the respective proportions in percentages. Data is shown as median and interquartile range. Mann-Whitney U test was used to analyze differences between uninfected and infected participants and sequential time points. A two-tailed p<0.05 is considered a significant difference. *p<0.05; **p<0.01.

To determine the cytotoxic CD8+ T cells responses in the vaccinees, SARS-CoV-2 S- and N-specific CD8+ T cell responses and the distribution of stimulated CD8+ T cells into memory cell subsets was analyzed (**Figure 8**). Stimulated CD8^+^ T cells specific to S or N peptides were characterized by co-expression of activation markers CD69 and CD137. The gating strategy is illustrated in **Figure 8A**; **Supplementary Figure 6**. Overall, both the S- and N-specific stimulation levels of CD8^+^ cells were lower compared to those of CD4^+^ cells (**Figure 8B**). Post the third vaccine dose, a low number of uninfected vaccinees exhibited CD8^+^ T cell response to the S-wt stimulation: 48% (23/48) at 3D3wk, 33% (5/15) at 3D3mo, and 55% (6/11) at 3D6mo, with the GM of SIs 1.6, 1.4, and 1.8, respectively (**Figures 8B, C**). At eight to twelve months after the third dose, only 25% (5/20) of the uninfected vaccinees exhibited a positive CD8^+^ T cell response to the stimulation with S-wt peptide pool. S-wt-specific CD8+ responses were not increased after the fourth vaccine dose, and 38% (5/13) and 42% (5/12) of the uninfected vaccinees showed a positive response at 4D3wk and 4D3mo, respectively.

In contrast to uninfected vaccinees, S-specific CD8+ responses were observed in 67% (20/30) of the infected vaccinees in at least one time point, and post the third vaccine dose at 3D8-12mo a significant difference was observed compared to uninfected vaccinees (p=0.0107; **Figure 8B**). However, the levels of S-wt-specific CD8+ T cells varied between the time points, and at 4D3wk only one infected vaccinee (1/8; 13%) had a positive response for S-wt-specific CD8+ T cells. Similar to N-specific CD4+ T cells, N-specific CD8+ responses remained low both in uninfected and infected vaccinees. Of the infected vaccinees, 50% (15/30) had N-specific CD8+ T cells in at least one time point (**Figure 8B**). The S-peptide responses in uninfected and infected vaccinees were consistent in stimulations with the wt and the Omicron BA.5 and XBB.1.5 peptide pools (**Figure 8D**). Interestingly, many Omicron infected vaccinees (11/30; 37%) who showed no S-wt-specific CD8+ T cells had a response in BA.5 and/or XBB.1.5-stimulated CD8+ T cells.

Memory cell subsets of S-wt-specific CD8+ T cells were predominantly of Temra cells, with smaller proportions of Tem and naïve cells (**Figures 8E, F**). These proportions were comparable between uninfected and infected vaccinees and remained relatively stable. However, at 4D3wk, both uninfected and infected vaccinees showed a minor increase in S-wt-specific naïve cells and a modest decrease in Temra cells.

### 3.7 Secretion of IFN-γ from the SARS-CoV-2 S- and N-peptide stimulated PBMCs

**Figure 9:**
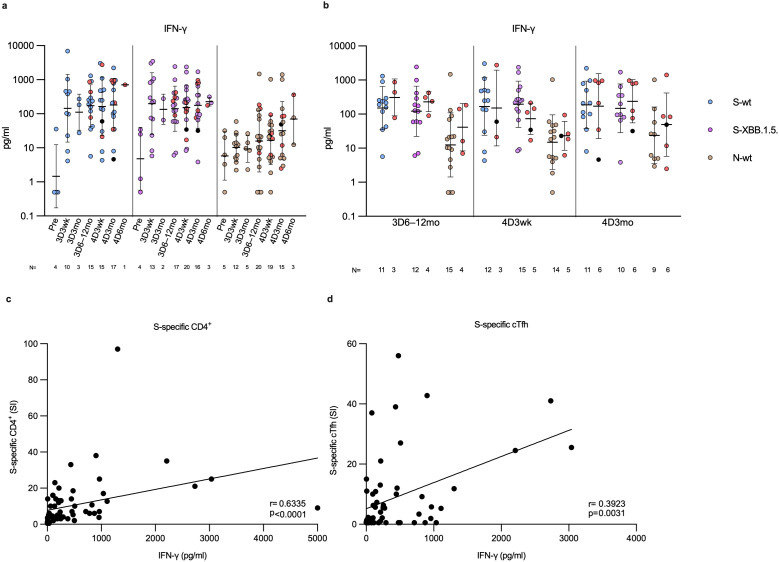
IFN-γ secretion in SARS-CoV-2 S and N peptide pool-stimulated PBMCs. **(A)** Levels of secreted IFN-γ (pg/ml) following PBMC stimulation with wild type spike (S-wt, blue dots) or N-specific (N-wt, green dots) peptide pool, or XBB.1.5 spike-specific (S-XBB.1.5, lilac dots) peptide pool. The PBMCs were collected from 26 participants before the vaccinations (Pre) and at three weeks, and three, and six to twelve months post the third vaccine dose (3D3wk, 3D3mo and 3D6–12mo, respectively), and three weeks, and three and six months post the fourth dose (4D3wk, 4D3mo and 4D6mo, respectively). **(B)** Vaccinees with breakthrough infection(s) (red and black dots) separated from uninfected vaccinees at each time point with each stimulant. Data is shown as geometric means and geometric standard deviations. In panel **(A)** the Kruskal-Wallis test followed by Dunn’s multiple comparisons test was used to determine the differences between time points within variant-specific groups, and in **(B)** the Mann-Whitney U test was used to analyze differences between uninfected and infected participants in different time points. A two-tailed p<0.05 is considered a significant difference. **(C)** The correlation of the levels of secreted IFN-γ (pg/ml) from the S-wt-stimulated PBMCs to SI values of S-wt-specific CD4+ T cell responses was calculated using nonparametric Spearman correlation analysis. **(D)** Nonparametric Spearman correlation analysis of the correlation of the levels of secreted IFN-γ (pg/ml) from the S-wt-stimulated PBMCs to SI values of S-wt-specific circulating follicular T-helper cells (cTfh).

To assess the activation and functionality of immune cells, PBMCs from a subset of four times vaccinated participants (N=26) were stimulated with spike peptides and analyzed for the secretion of the CD4+ and CD8+ effector cytokine IFN-γ (**Figure 9**). At all-time points after vaccination, following stimulation with both wt and XBB.1.5 S-peptide pools, IFN-γ was secreted at steady levels, with no significant differences between the time points of sample collection (**Figure 9A**). Similar to AIM expression, IFN-γ levels were comparable between uninfected and infected vaccinees (**Figure 9B**). Overall, IFN-γ levels were lower after stimulation with N-specific peptides compared to S-wt- and S-XBB.1.5-specific peptides, with significant differences at time points 3D3wk and 4D3wk (**Supplementary Figure 8A**). In addition, the secretion of IFN-γ following stimulation with N-specific peptide pool did not differ from the DMSO stimulated secretion of IFN-γ (**Figure 9A**; **Supplementary Figure 8A**). A statistically significant correlation (r=0.6335, p<0.0001) was observed between S-wt peptide activated CD4+ T cells and secreted IFN-γ levels from S-wt-stimulated PBMCs (**Figure 9C**). Furthermore, a weak positive correlation was evident between S-wt peptide activated cTfh cells and secreted IFN-γ levels from S-wt-stimulated PBMCs (r=0.3923, p=0.0031, **Figure 9D**). These results confirm that vaccine- and infection-induced SARS-CoV-2-specific T cells are functional at least up to one year after immunization.

## 4 Discussion

Most of the global disease burden during the COVID-19 pandemic has been associated with Omicron variants such as BA.1, BA.2, BA.5, BQ.1 (BA.5 derived), and XBB (BA.2 derived) and their subvariants (20). A characteristic feature of the Omicron variants has been the ability of the virus to evade COVID-19 vaccine- and infection-induced neutralizing antibodies, rendering the population vulnerable to infection with emerging variants (21–23).

In this longitudinal follow-up study, we analyzed the humoral and cell-mediated immunity induced by up to four COVID-19 vaccine doses and Omicron breakthrough infections. This work is an extension to our ongoing study (15, 16, 18, 24) of COVID-19 vaccinated HCWs to cover a longer follow-up time of up to 12 months after the third vaccine dose. We also analyzed the strength and variant-specificity of the immune responses after a fourth vaccine dose or a breakthrough infection(s). During the follow-up over 70% of the vaccinees contracted one or more Omicron variant infections representing the high transmission of Omicron variants in Finnish population since 2022 (25).

In Finland, the fourth vaccine dose is recommended for risk groups [mainly age-based; persons aged 65 and over (26)] who generally have weaker vaccine-induced antibody responses (16, 18, 27). In our study cohort, only five participants were over 65 years old at the time of the fourth vaccine dose and most of the participants receiving the fourth vaccine dose belonged to other medical risk groups. The third and fourth vaccine doses increased S1-specific antibodies to high levels, and the waning of binding as well as neutralizing antibodies was slower after the fourth dose, indicating that the repeated vaccination delayed the disappearance of S1-specific antibodies. Omicron breakthrough infection further induced and maintained somewhat higher IgG antibody levels and memory B cell responses as compared to uninfected individuals, the infection being as an additional booster dose. In contrast, anti-N IgG antibody levels and memory B cell responses were low in infected vaccinees. Likely the vaccine primes the antibody response more towards S-specific response and also the N-specific antibodies seem to wane quickly (28, 29). Either way, a weak N-specific response may question the value of detection of anti-N antibodies as an indicator of a SARS-CoV-2 infection.

Neutralizing antibodies are to date the best correlate of protection against severe COVID-19 (30, 31). Here, we analyzed the neutralizing antibodies against Omicron variants BA.2, BA.5, and XBB.1.5. Compared to three vaccine doses, the fourth vaccine dose induced slightly better immunity against the more recent XBB.1.5 variant. However, after the fourth vaccine dose the levels of neutralizing antibodies increased to similar levels as after the third vaccine dose, indicating that additional booster vaccinations restore but do not override the antibody levels. Interestingly, the bivalent vaccine induced equally high neutralizing antibodies against D614G as the monovalent vaccine, and repeated vaccinations with the original Wuhan-type monovalent vaccine or booster vaccination with a bivalent BA.1 or BA.4/5 vaccine did not broaden the specificity of neutralizing antibodies against XBB.1.5. These results indicate that the vaccines elicit antibody responses based on immune imprinting and the repeated Omicron exposure does not override ancestral SARS-CoV-2 immune imprinting. This observation is in line with previous studies (22, 23, 32) showing that the bivalent BA.4/5 vaccine induces cross-reactive antibodies to ancestral SARS-CoV-2 and provides no advantage over the original vaccine against emerging variants. On the other hand, an Omicron breakthrough infection elicited higher levels of neutralizing antibodies against the Omicron variants, especially against XBB.1.5, than the fourth vaccine dose with a monovalent Wuhan-type or bivalent BA.1 or BA.4/5 vaccine, underlining the fact that a breakthrough infection by an Omicron variant induces a broad neutralization response (33, 34). The data on neutralization capacity of elicited antibodies indicates that the bivalent BA.4/5 was not as effective as initially anticipated (35, 36). However, the first studies on the XBB.1.5 vaccine suggest that a monovalent, variant-specific booster dose induces good immune responses also against more recent Omicron variants (37, 38) although this does not completely alleviate vaccine-induced immune imprinting (39).

To estimate the duration of antigen specific humoral immunity, it is important to assess the presence and functional activity of circulating S protein-specific memory B cell in vaccinees. Here we quantitated with ELISpot the circulating memory B cells able to differentiate into functional S1-specific antibody-secreting B cells in vaccinees with and without breakthrough infection. Consistent with the results by others (40, 41), we showed increase in circulating S1-specific memory B cells after vaccinations or infections, and correlation of the higher serum antibody levels with the higher levels of circulating S1-specific memory B cells, indicating that both vaccinations and infections expand the memory B cell compartment.

In our analysis of cell-mediated immunity, we used activation induced marker (AIM) assay to identify S- and N-specific T cells and to further type the cells to different memory T cell subtypes. The strongest activation in vaccinees was seen in S-specific CD4+ T cells. In addition, a breakthrough infection induced the formation of N-specific CD4+ T cells. Consistent with other studies (42, 43), S-specific CD4+ T cells exhibited mainly effector and central memory phenotypes. Together these results indicate that *de novo* T cells can be recruited after exposure to additional T cell epitopes through vaccinations and infections, and that COVID-19 vaccine-induced T cell responses can be recalled at least a year after vaccination. In contrast, CD8+ cells stimulated by S peptide pools had weaker and more variable responses than those seen in CD4+ T cells. Further analysis of circulating follicular T helper cells (cTfh), which are important for B cell activation and germinal center responses (44), showed that S-specific cTfhs were detected after vaccination and infection. Although cTfhs have been shown to correlate with antibody levels during acute infection (45), we did not see any correlation between the frequency of cTfh response and anti-S IgG antibody levels after the booster vaccinations. This may be because our vaccinees had been vaccinated several times and thus the antibody responses were dominated by B cell memory responses rather than the formation of new B cell clones. It is noteworthy that we used peptides pools covering the whole S protein and thus the amino acid substitutions, even in the XBB.1.5 variant, represent a minority of the whole S protein. S-specific T cells were functionally active since they readily produced and secreted IFN-γ, the typical Th1 type marker cytokine. Our results on cell-mediated immune responses indicate that the spike-specific T cell responses induced by COVID-19 vaccines are retained at least for 12 months after the last vaccine dose, cross-react with SARS-CoV-2 variants, and are consistently maintained at stable levels, irrespective of vaccine booster doses or breakthrough infections. We did not observe an increase in T cell responses after repeated vaccinations or infection; and importantly, we did not observe a decrease or an exhaustion of T cell responses after four vaccine doses and a natural infection(s).

This study provides a comprehensive analysis of humoral and cell-mediated immunity induced by repeated COVID-19 vaccinations followed by an Omicron breakthrough infection. Our data shows that the fourth vaccine dose readily boosts the humoral immune responses and slows down the decay of antibody levels. While the vaccinations and the infection efficiently induce serum anti-S IgG antibody levels, these antibodies decline with a half-life of 3-4 months. The decline in serum antibodies may render the vaccinees susceptible to infection when longer time has passed since the last vaccination or infection. A reassuring feature in the immune responses is that ancestral vaccine-induced T cell responses are very well retained providing protection against a severe disease. Altogether, our data indicates that repeated vaccination does not lead to an exhaustion of T cell responses, nor does it further broaden the antibody response. Thus, booster vaccinations should be updated to match the circulating variants to limit the harm caused by multiple SARS-CoV-2 infections.

## 5 Limitations of the study

Limitations of the study include the small sample size after the fourth vaccine dose and in memory B and T cell analysis. Males were underrepresented and the comparisons between the time points involved different number of participants and were not controlled for age, potentially affecting the antibody responses. Immune responses after the third and fourth vaccine doses were not directly comparable due to the difference in age and health status. In addition, some Omicron infections were likely unreported since SARS-CoV-2 testing is infrequent and not all vaccinated individuals with breakthrough infections produce detectable anti-N antibodies. Lastly, CD8+ T cell responses may have been impacted by using longer peptides (15-mers) that preferentially bind to HLA class II molecules rather than HLA class I molecules (46).

## Data Availability

The original contributions presented in the study are included in the article/**Supplementary Material**, further inquiries can be directed to the corresponding author/s.
