# Supplementary material for: Long-term COVID-19 vaccine- and Omicron infection-induced humoral and cell-mediated immunity
Source: Front Immunol. 2024 Nov 21;15:1494432. doi: 10.3389/fimmu.2024.1494432 (PMC11617562; doi:10.3389/fimmu.2024.1494432)
Supplement: Supplementary file 1 [file DataSheet1.pdf]

Supplementary data

Supplementary Table 1. SARS-CoV-2 S1- and N-specific IgG antibody levels and fold changes in HCWs after the first, second, third and fourth vaccine doses in uninfected HCWs. The geometric means (GM) and geometric standard deviation factors (GSD) of the SARS-CoV-2 S1- and N-specific IgG antibody levels three weeks and three months after the first (1D3wk and 1D3mo, respectively), second (2D3wk and 2D3mo, respectively), third (3D3wk and 3D3mo, respectively), and fourth vaccine dose (4D3wk and 4D3mo, respectively) are shown. The geometric means of the antibody levels are compared to the previous sample (third row) or to the corresponding sample from the previous three weeks or three months post vaccine dose (bottom row, i.e. fold change in 1D3wk GM compared to 2D3wk GM is 4.8x).

	**1D3wk***<br>N=281	**1D3mo***<br>N=151	**2D3wk***<br>N=280	**2D3mo***<br>N=285	**3D3wk**<br>N=93	**3D3mo**<br>N=71	**4D3wk**<br>N=26	**4D3mo**<br>N=23
**Anti-S1 IgG** (EIA-units)<br>GM<br>GSD	 23<br>4	 10<br>3	 120<br>2	 70<br>2	 118<br>1	 85<br>1	 119<br>1	 109<br>1
**Anti-N IgG** (EIA-units)<br>GM<br>GSD	 2<br>2	 2<br>2	 2<br>2	 2<br>2	 1<br>2	 1<br>2	 1<br>2	 1<br>2
**Fold change in anti-S1 IgG** compared with previous sample GM		0.4x	12.0x	0.6x	1.7x	0.7x	1.4x	0.9x
**Fold change in anti-S1 IgG** compared with corresponding previous dose GM			5.2x		1.0x		1.0x	
				7.0x		1.2x		1.3x

*Data from our previous studies [19,26]

Supplementary table 2. Fluorochrome-conjugated anti-human antibodies used in AIM assay.

Anti-human antibody	Fluorochrome	Clone	Manufacturer	Amount/test (µg)	Catalogue number
CD45	APC-eFluor780	HI30	Invitrogen	0.05	47-0459-42
CD3	eFluor506	UCHT1	Invitrogen	0.5	69-0038-42
CD4	eFluor450	RPA-T4	Invitrogen	0.25	48-0049-42
CD8a	PerCP-eFluor710	SK1	Invitrogen	0.125	46-0087-42
CD69	PE	FN50	BD Biosciences	0.03	555531
CD134	PE/Cyanine7	Ber-ACT35	BioLegend	2.0	350012
CD137	APC	4B4-1	BioLegend	0.5	309810
CD45RA	Brilliant Violet 785	HI100	BioLegend	0.1	304140
CD197 (CCR7)	PE/Dazzle 594	G043H7	BioLegend	0.75	353236
CD185 (CXCR5)	Brilliant Violet 605	J252D4	BioLegend	0.7	356930

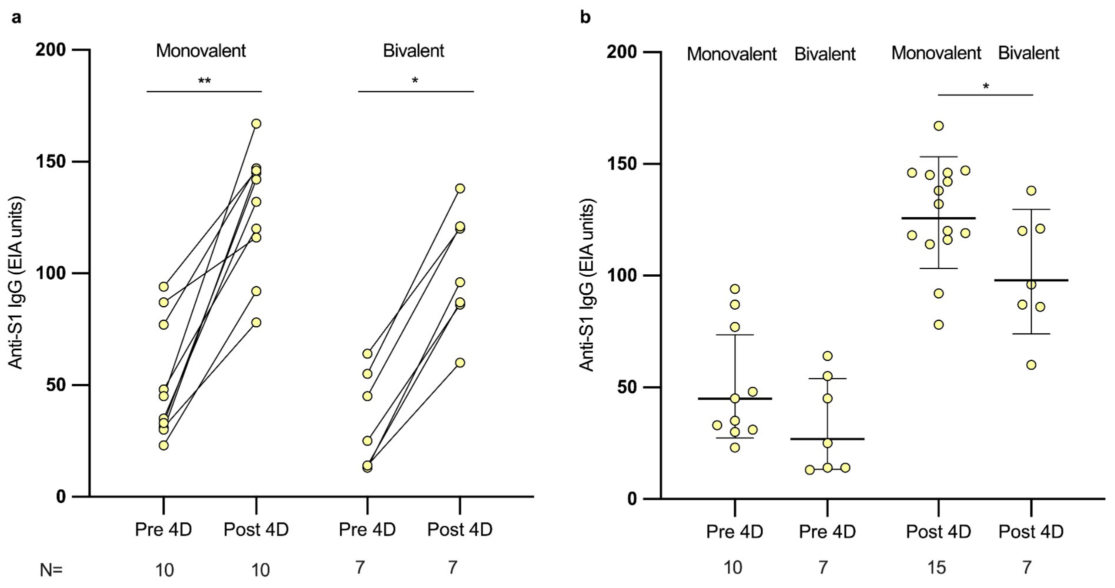

Figure S1. SARS-CoV-2 S1-specific IgG antibody responses in HCWs vaccinated with a monovalent or bivalent COVID-19 mRNA-vaccine as the fourth dose. a–b Anti SARS-CoV-2 S1-specific IgG antibody responses measured before (Pre 4D) and after (Post 4D) the fourth vaccination in uninfected HCWs who received the monovalent (Moderna; N=3 or Pfizer; N=12) or bivalent (Pfizer BA.1; N=2 and BA.4/5; N=5) COVID-19 vaccine as the fourth dose. **a** Responses shown in individual vaccinees, and **b** in groups of vaccinees. Geometric mean with geometric SD is shown. Samples with no data on both data points were excluded from the comparison between time points and Wilcoxon signed rank test was used to measure statistical significance for the paired samples. Mann-Whitney U test was used to measure statistical significance between the vaccination groups within time points. Two-tailed p-values <0.05 were considered statistically significant. *p<0.05; **p<0.01

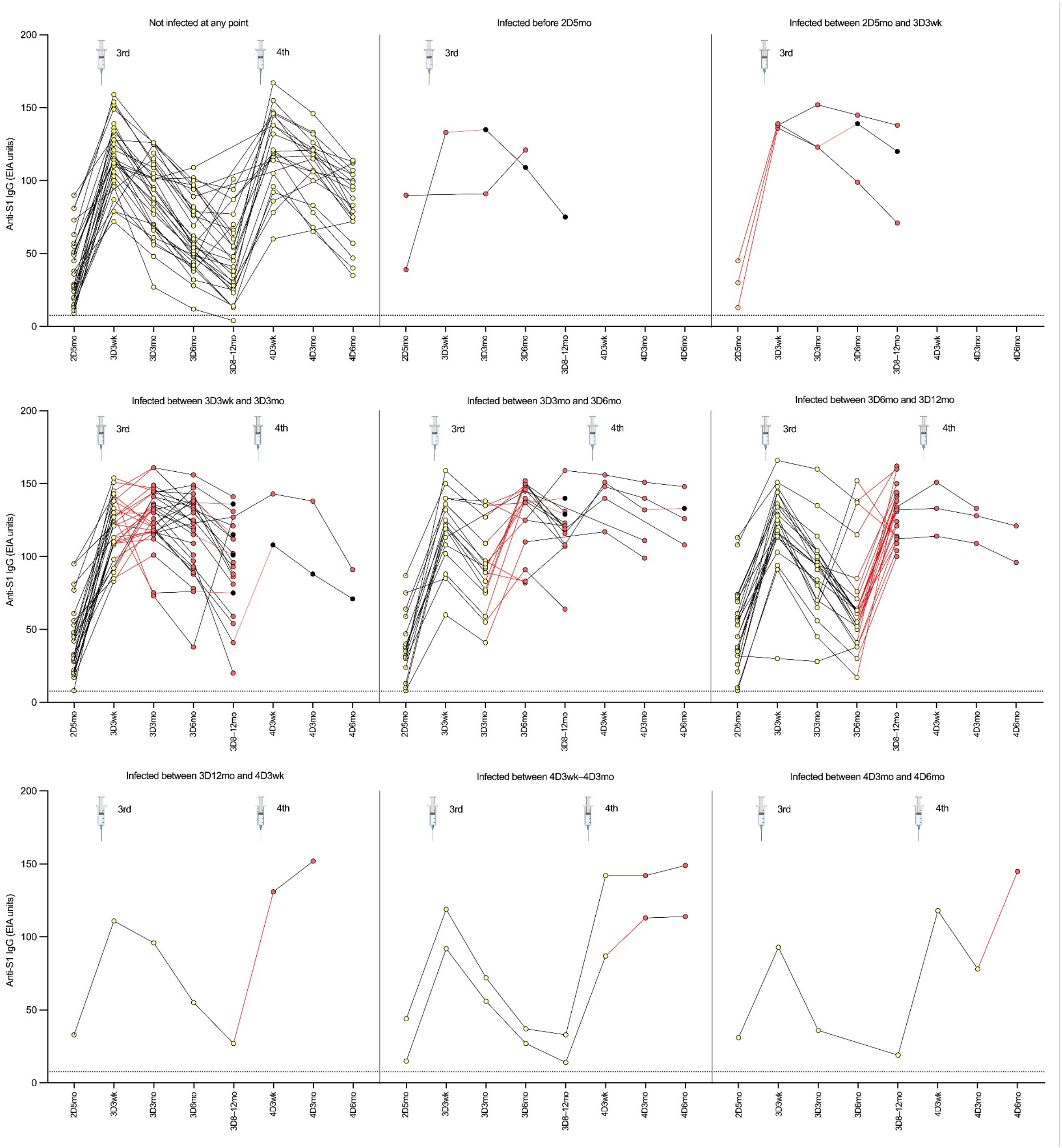

Figure S2. SARS-CoV-2 S1-specific IgG antibody responses in three and four times vaccinated HCWs separated into groups based on the time of a breakthrough infection. Vaccinees are separated into nine groups based on the time of a breakthrough infection to display the effect of the infection and vaccination on the kinetics of the SARS-CoV-2 S1-specific IgG antibody levels. Individuals with two breakthrough infections are grouped based on the timing of their first infection. Red lines indicate the timing of the breakthrough infection, red dots represent samples collected after one breakthrough infection, black dots represent samples collected after two breakthrough infections, and dashed lines indicate the cut-off value.

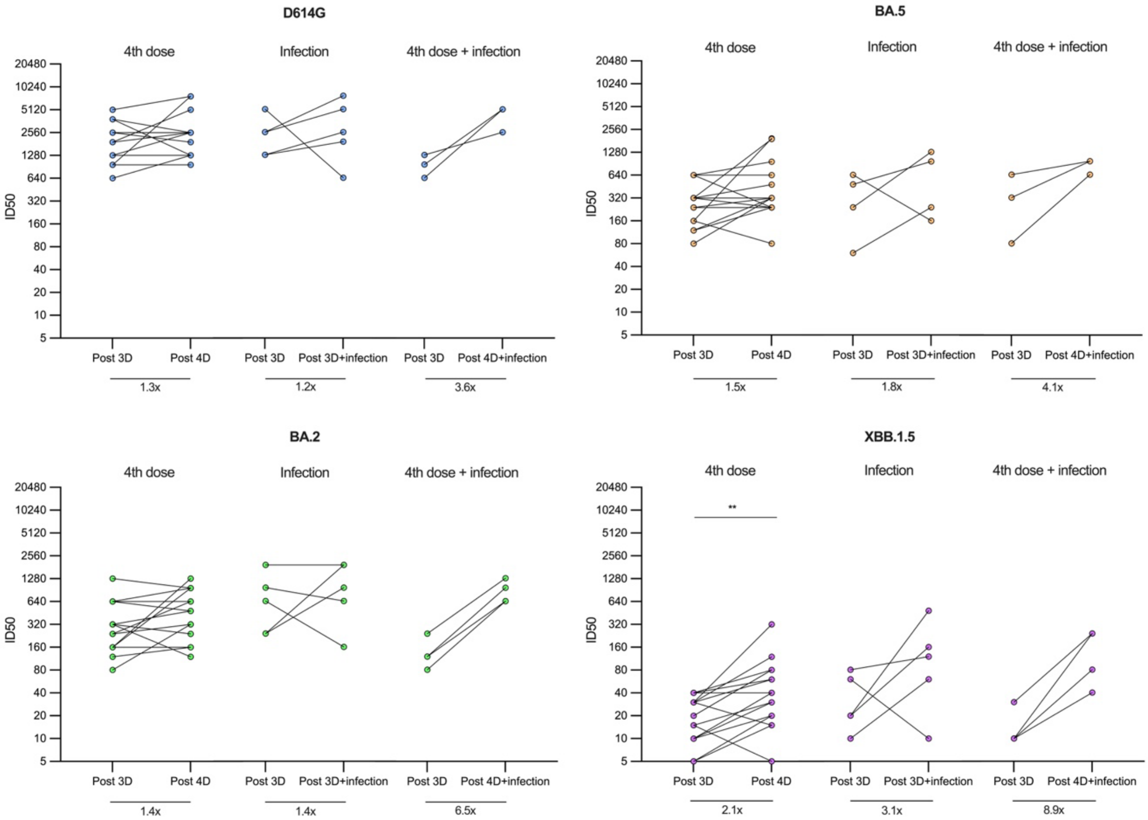

Figure S3. Neutralizing antibody titers before and after the fourth vaccine dose and/or a breakthrough infection. Neutralizing antibody titers against SARS-CoV-2 variants before (Post 3D) and after the fourth vaccine dose (Post 4D, N=17) or Omicron breakthrough infection alone (Post 3D+infection, N=5), or after the fourth dose and a breakthrough infection (Post 4D+infection, N=4). All the Post 3D-samples in all three groups of vaccinees were collected three weeks post the third dose. The rest of the samples were collected three weeks post the fourth dose (Post 4D), eight to twelve months post the third dose (Post 3D+infection) and three weeks to six months post the fourth dose (Post 4D+infection). The fold changes are counted from the geometric means of the antibody levels in each timepoint. Wilcoxon signed rank test was used to analyze the statistical significance between the time points. Two-tailed p-values <0.05 were considered statistically significant. **p<0.01

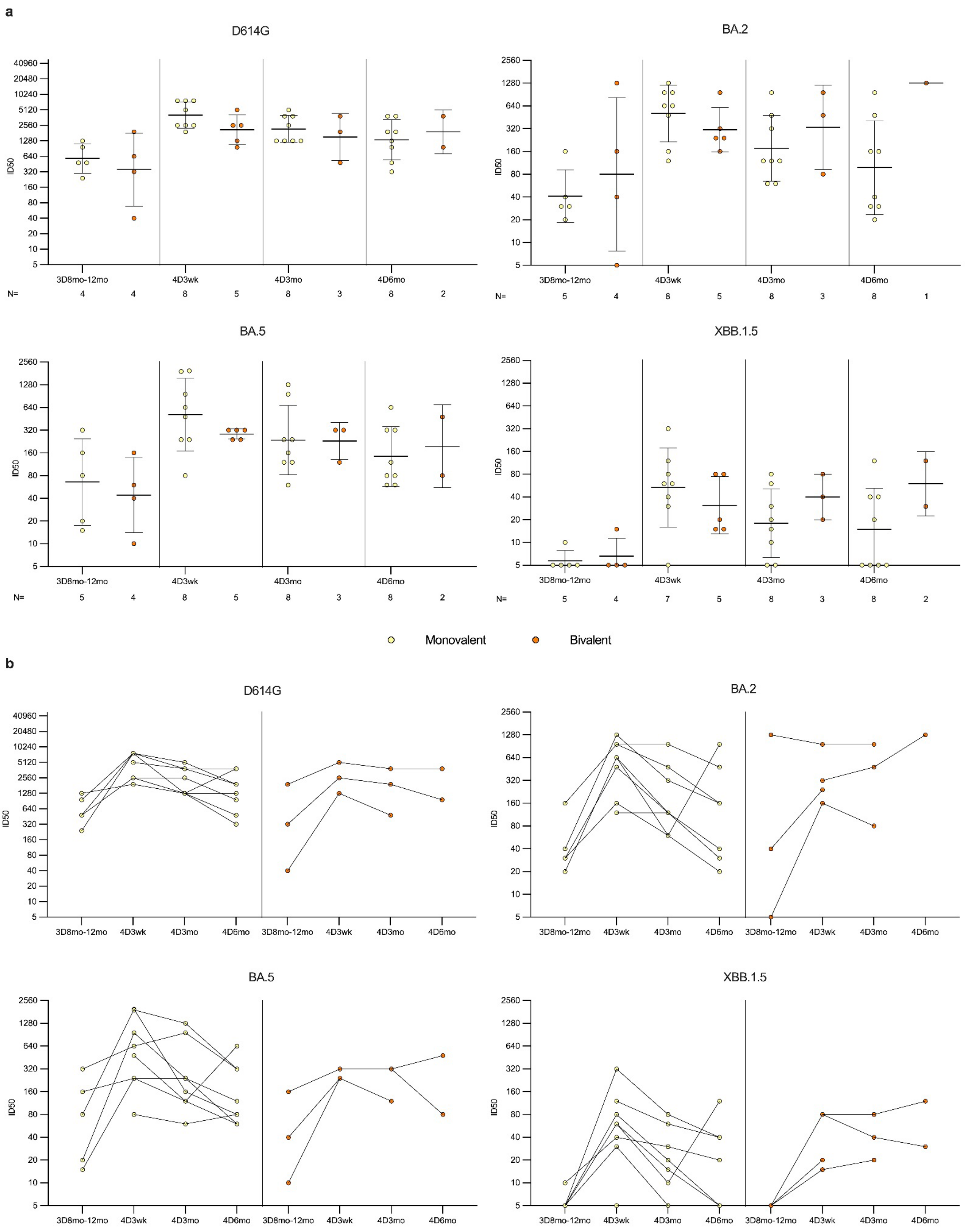

Figure S4. Neutralizing antibody titers of uninfected HCWs vaccinated with a mono- or bivalent vaccine as the fourth dose. Neutralizing antibody titers in the sera of uninfected HCWs vaccinated with a monovalent (Pfizer or Spikevax, yellow dots) or a bivalent (BA.1 or BA.4/5, orange dots) vaccine as the fourth dose. Samples were collected eight to twelve months (3D8–12mo; N=5 monovalent and N=4 bivalent vaccine) after the third vaccine dose, three weeks (4D3wk; N=8 monovalent and N=5 bivalent vaccine), and three months (4D3mo; N=8 monovalent and N=3 bivalent vaccine) and six months (4D6mo; N=8 monovalent and N=2 bivalent vaccine) after the fourth vaccine dose. Titers are shown as **a** groups of vaccinees, and **b** followed up as individual vaccinees. Geometric means and

geometric standard deviations of the antibody levels are shown. Mann-Whitney U-test was used to analyze the statistical significance between the samples collected from uninfected and infected vaccinees within time points, and between the samples collected at three weeks, and three and six months after the third and fourth vaccine dose. Two-tailed p-values $p < 0.05$ were considered statistically significant.

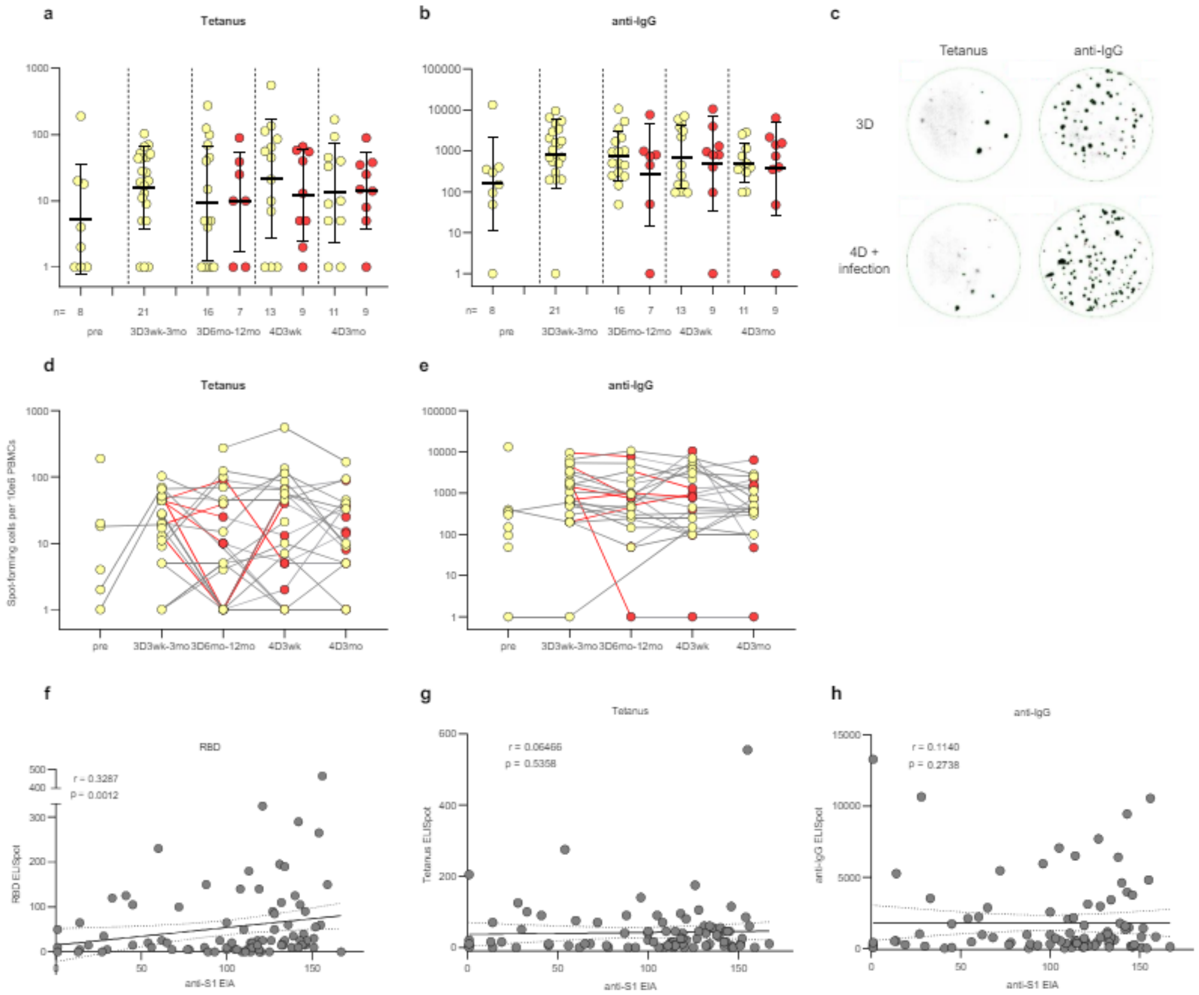

Figure S5. Tetanus toxoid and anti-IgG specific memory B cell responses in vaccinees. PBMCs collected before COVID-19 vaccination (pre, N=8), three weeks or twelve months after the third vaccine dose (3D3wk, N=21; 3D12mo, N=23), and three weeks or three months after the fourth vaccine dose (4D3wk, N=22; 4D3mo, N=20) were stimulated with IL-2 and R848. ELISpot was used to detect **a,d** the tetanus toxoid (tet)-specific and **b,e** IgG-productive (anti-IgG) memory B cells capable of turning into antibody-secreting cells. Red dots indicate the spot-forming cells after SARS-CoV-2 infection, and red lines the interval during which a breakthrough infection occurred. **c** Visual representation of the spots formed by memory B cells specific to tetanus toxoid and by IgG-productive memory B cells in individuals who received three vaccine doses, or four vaccine doses and experienced a breakthrough infection. **f–h** Nonparametric Spearman correlation analysis of the SARS-CoV-2 RBD-specific (RBD ELISpot), tetanus toxoid-specific (Tetanus ELISpot), and IgG-specific (anti-IgG ELISpot) memory B cell responses to S1-specific IgG antibody responses (anti-S1 EIA). Statistical significance was determined using the Mann-Whitney U test. Statistically significant differences (panels a and b) were not found.

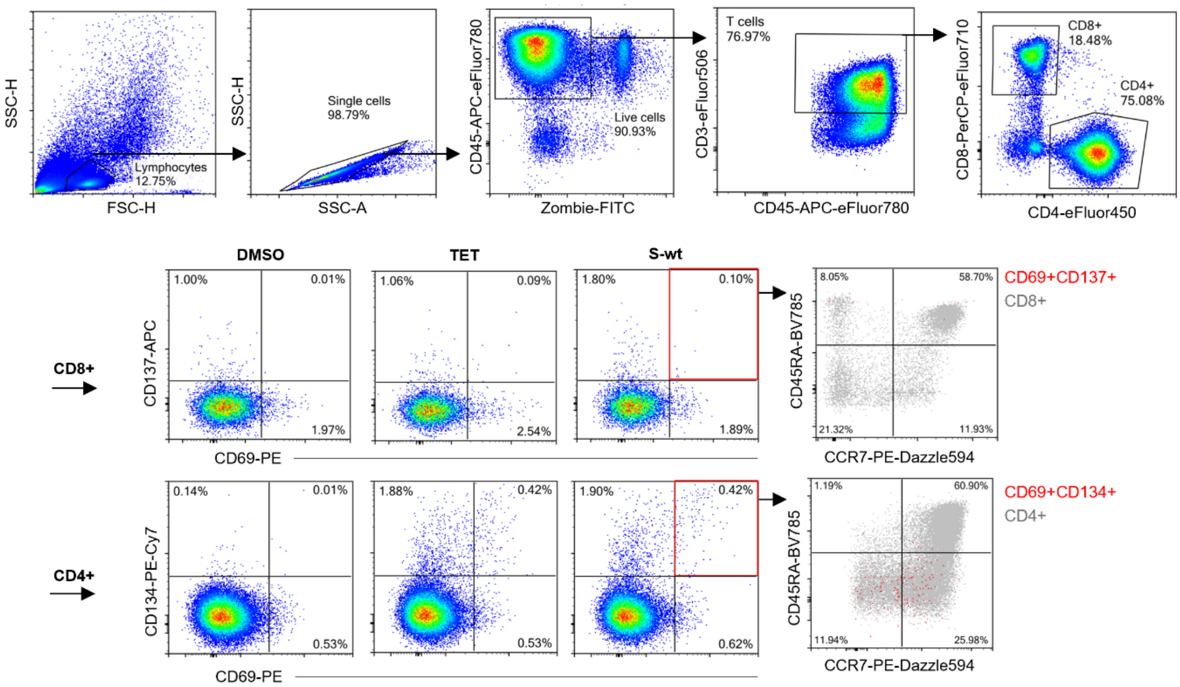

Figure S6. Gating strategy for SARS-CoV-2 S-specific CD4+ and CD8+ T cell populations in peripheral blood mononuclear cells (PBMCs). To activate CD4+ T cells, PBMCs were stimulated with a SARS-CoV-2 spike peptide pool (S-wt). DMSO was used as a negative control, and tetanus toxoid (TET) as a positive control. Plots show the representative data from one vaccinee with three vaccine doses and an Omicron breakthrough infection.

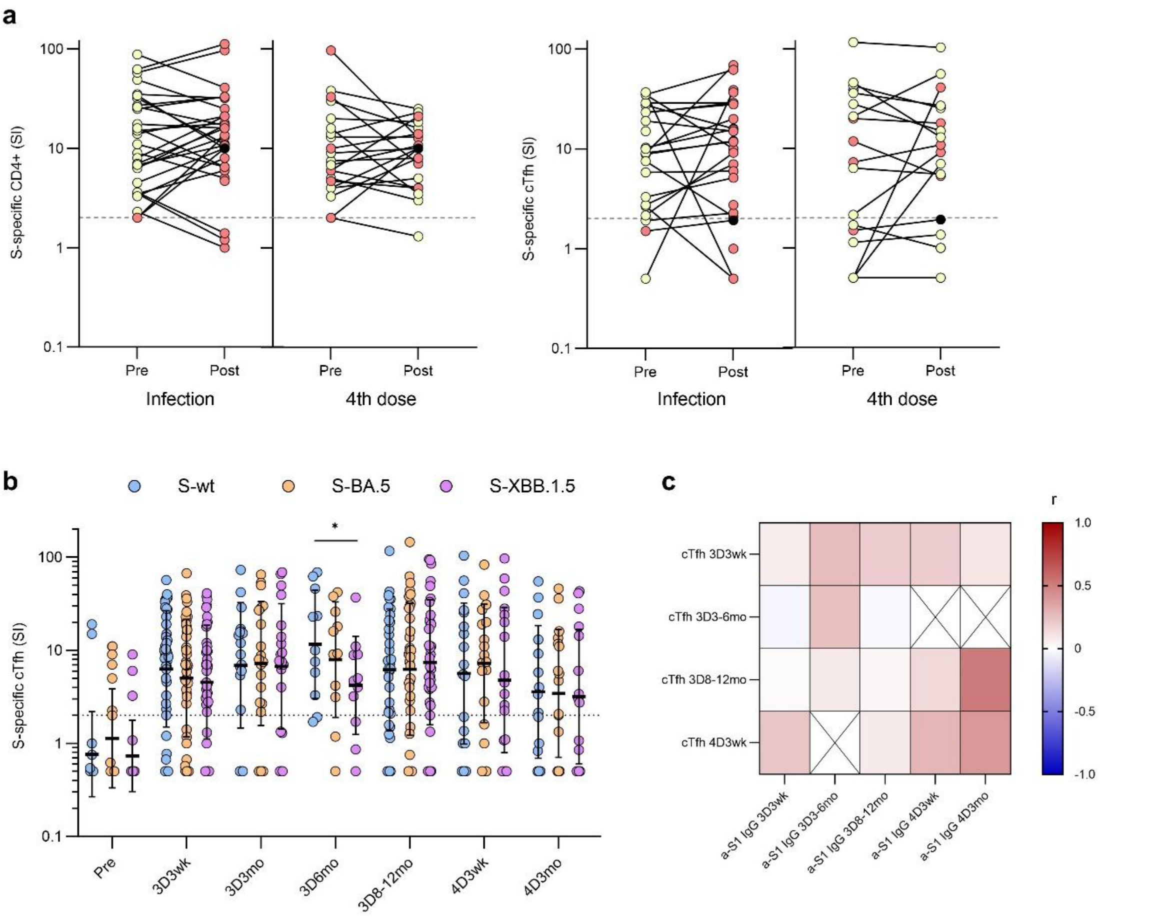

Figure S7. S-wt-specific cTfh responses and the correlation with anti-S1 IgG responses. **a** S-wt-specific cTfh responses before and after an Omicron breakthrough infection (red dots) or the fourth vaccine dose. Wilcoxon test was used to analyze differences between the time points. **b** Comparison of the S-specific cTfh responses at different time points after stimulation with S peptide pools from wild type (S-wt) and Omicron variants BA.5 and XBB.1.5 (S-BA.5 and S-XBB.1.5). Statistical differences were analyzed with the Friedman test followed by Dunn's multiple comparison test. Two-tailed p-values <0.05 were considered statistically significant. *p<0.05. **c** Spearman correlation of S-wt-specific cTfh frequency and anti-S1 IgG antibody levels. All correlations were non-significant.

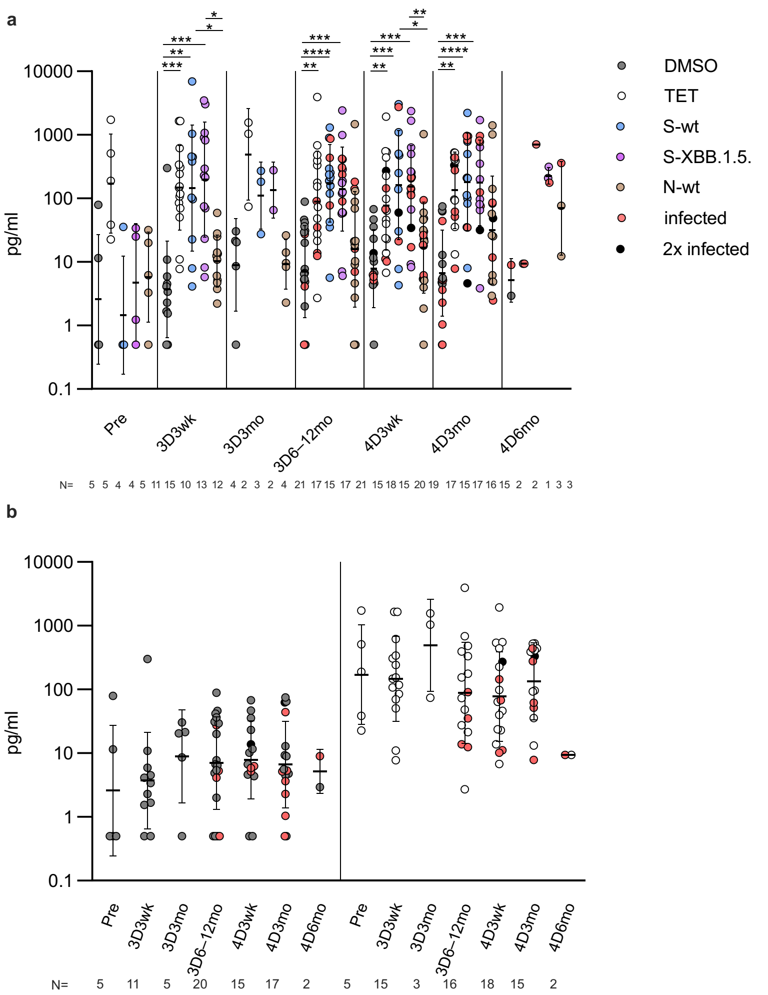

Figure S8. IFN-γ secretion from PBMCs stimulated with S- or N-specific peptide pools. **a** IFN-γ levels (pg/ml) in supernatants following stimulation of PBMCs with DMSO (blue dot), tetanus toxoid (TET, white dot), wild type spike (S-wt, blue dot), N-specific (N-wt, green dot), and XBB.1.5 spike-specific (S-XBB.1.5, lilac dot) peptide pools. PBMCs were obtained from 26 participants both before the vaccination (Pre) and at three weeks (3D3wk), three months (3D3mo), and six to twelve months (3D6–12mo) post the third vaccine dose, and three weeks (4D3wk), three months (4D3mo), and six months (4D6mo) post the fourth vaccine dose. **b** The levels of secreted IFN-γ (pg/ml) following stimulation of PBMCs with DMSO or tetanus toxoid (TET) are shown separately. **a–b** Red dots represent samples collected from vaccinees with a SARS-CoV-2 breakthrough infection and black dots represent samples collected from vaccinees with two (or more) breakthrough infections. Data is shown as geometric means and geometric standard deviations of the means. Kruskal-Wallis test followed by Dunn's multiple comparisons test was used to determine the differences between variant-specific groups within each time point and between time points within variant-specific groups. A two-tailed $p<0.05$ is considered a significant difference. *$p<0.05$; **$p<0.01$; ***$p>0.001$; ****$p<0.0001$.
